# Postnatal plasticity in the paralaminar nucleus of the pallial amygdala in juvenile swine brain

**DOI:** 10.1007/s00429-026-03106-8

**Published:** 2026-04-06

**Authors:** Júlia Freixes, Ester Desfilis, Loreta Medina

**Affiliations:** 1 Department of Medicina Experimental, Universitat de Lleida, Lleida, Spain; 2 Laboratory of Evolutionary Developmental Neurobiology, Lleida’s Institute for Biomedical Research-Dr. Pifarré Foundation (IRBLleida), Lleida, Spain

**Keywords:** Postnatal neurogenesis, Gyrencephalic brain, Migratory streams, Neuroplasticity, Amygdalar pallium, Paralaminar nucleus, Intercalated amygdalar cells, Pig

## Abstract

The aim of this study was to investigate the presence of immature cells and their phenotype in the pallial amygdala of juvenile swine brains by way of immunoreactivity for the microtubule-associated protein doublecortin (DCX), combined with the cell proliferation marker Ki-67 and different neuronal markers, including NeuN and the transcription factor COUP-TFII (NR2F2, critical for amygdalar development and adult phenotype maintenance). Our results showed the existence of numerous DCX+ cells along the external border of the basal amygdalar complex, adjacent to the amygdalar capsule, in an area identified as the swine paralaminar nucleus. These cells showed a patchy distribution, with shell-like clusters of DCX+ cells partially surrounding islands of non-stained large cells of the basal amygdalar complex. The paralaminar nucleus and the other nuclei of the basal amygdalar complex also contained abundant neurons expressing COUP-TFII, but not the transcription factor FOXP2, which defines the clusters of intercalated amygdalar cells. Paralaminar patches of DCX+ cells were more abundant at posterior levels, where they were continuous with chains of DCX+ cells with migratory-like morphology that appear to come from the subventricular zone surrounding the temporal horn of the lateral ventricle. This part of the ventricular/subventricular zone (vz/svz) contained many cells coexpressing COUP-TFII/Ki-67 and DCX/Ki-67, suggesting that this might be the source of the immature cells found in the paralaminar nucleus. Overall, these results show that the swine pallial amygdala shows a prolonged postnatal plasticity and contains a reservoir of immature neurons mainly located in the paralaminar nucleus and related vz/svz, resembling the situation seen in humans. These findings also point to the swine as an excellent model to study mechanisms behind postnatal plasticity in the amygdala of gyrencephalic animals, and the role of this protracted plasticity in amygdala function and dysfunction.

**Supplementary Information:**

The online version contains supplementary material available at 10.1007/s00429-026-03106-8.

## Introduction

In humans and non-human primates, the amygdala shows a remarkably prolonged maturation, extending through adolescence and beyond, which may be related to the increasing involvement of this structure over time in social cognition and adaptation to changing social contexts (Chareyron et al. 2012; Avino et al. 2018). The protracted postnatal development of the amygdala involves the presence of a large number of immature neurons, especially concentrated in the paralaminar amygdalar nucleus (PL) (De Campo and Fudge 2012), which appears as an immature cell reservoir from where new neurons migrate, mature and incorporate into the adjacent basal and accessory amygdalar nuclei (Chareyron et al. 2012; Avino et al. 2018; Sorrells et al. 2019). Since the PL is a major target of hippocampal projections (Fudge et al. 2012), it has been proposed to be an interface linking autobiographical memories with emotional valence (Jin et al. 2015; Kim et al. 2017; discussed by Sorrell et al. 2019. In humans and monkeys, the PL increases in size and neuron number during juvenile ages, but this fails to occur in autism spectrum disorder (Avino et al. 2018).

In humans and other primates, immature neurons are abundant in PL of children and express microtubule-associated protein doublecortin (DCX), polysialylated neural cell adhesion molecule (PSA-NCAM) and B cell lymphoma 2 protein (BCL2) (Bernier et al. 2002; De Campo and Fudge 2012; Sorrells et al. 2019; Ghibaudi et al. 2025). Many of these immature neurons transition during adolescence into mature glutamatergic neurons that contain the vesicular glutamate transporter VGLUT2 (SCL17A6) and the transcription factors TBR1 and COUP-TFII (NR2F2), but not other transcription factors typical of the caudal ganglionic eminence such as SP8 (Sorrells et al. 2019). This suggests that these immature neurons originate in the pallium, but their exact spatial and temporal origin, either prenatally and/or postnatally, remain unknown or controversial (Bernier et al. 2002; Sorrell et al. 2019; Ghibaudi et al. 2025). Independent of their origin, the existence of large numbers of excitatory neurons in primate PL that remain immature for relatively long periods (decades in humans) indicates an extraordinary plasticity of the pallial amygdala (Sorrells et al. 2019; Ghibaudi et al. 2025), which function remains to be explored. Immature neurons are more abundant and persist during a longer postnatal period in the basal complex of the amygdala in large-brained, mostly gyrencephalic mammals (especially primates, but also carnivores as the cat and artiodactyls as the sheep), but they are scarce and decline rapidly in small-brained mammalian species (including rodents) (Piumatti et al. 2018; Alderman et al. 2024; Ghibaudi et al. 2025). In primates, immature neurons continue to be observed in the basal complex of the amygdala even in aged adults, while in rodents (like mouse) their density is much lower and there is a sharp decline in juveniles (Alderman et al. 2024; Ghibaudi et al. 2025). This is likely related to the very small PL found in rodents, difficult to distinguish from the intercalated cells (De Campo and Fudge 2012). Only recently, with the use of multiple fluorescent labeling and single cell transcriptomics, it was possible to reliably distinguish in mouse between PL (expressing DCX, COUP-TFII / NR2F2, and several glutamatergic markers) from the intercalated amygdalar cells (expressing FOXP2 and GABAergic markers) (Alderman et al. 2024).

In spite of the detailed knowledge on the distribution, phenotype and changes throughout ontogeny of amygdalar immature neurons in some primates and rodents, data in other species is still scarce and a putative paralaminar nucleus remains poorly defined. In recent years, the swine has emerged as a large-brained gyrencephalic species, which brain development and organization resemble more closely that of humans, compared to the murine brain (Lind et al. 2007; Kinder et al. 2019; Liu et al. 2021; Ghibaudi et al. 2025). Moreover, it shows abundant levels of DCX+ immature neurons throughout all olfactory areas in piglets and juveniles, which appear to migrate from different rostrocaudal levels of the subventricular zone of the lateral ventricle (SVZ) and/or the adjacent white matter (Costine et al. 2015; Torrijos-Saiz et al. 2025; Freixes et al. 2025). While the SVZ is usually known as a source of GABAergic interneurons for the olfactory bulb (Alvarez-Buylla and García-Verdugo 2002; Kohwi et al. 2005, 2007; Young et al. 2007), studies in different mammalian species including swine show that it also gives rise to glutamatergic neurons that migrate through the rostral migratory stream and the external capsule to different olfactory structures of the piriform lobe (Freixes et al. 2025). DCX+ immature neurons were also found in the olfacto-recipient cortical area of the amygdala in juvenile swine, but their presence in the basal amygdalar complex and the existence of a PL is unexplored in swine. An excellent approach to help with the identification of homologue brain structures is by studying their developmental origin, as this is a good reference for understanding their topological position, which remains identical throughout ontogeny and in evolution (Nieuwenhuys and Puelles 2016). In primates, the basal amygdalar complex, including the PL, was proposed to derive from the so-called “inferior ganglionic eminence”, adjacent to the temporal horn of the lateral ventricle (tlv), and postnatally the PL resides in the same location adjacent to the former inferior ganglionic eminence (reviewed by De Campo and Fudge 2012). The developmental origin of the basal complex and cortical areas of the amygdala has recently been reformulated in mouse, and they appear to derive from a distinct posteroventral ventricular sector of the telencephalon, named the amygdalar pallium (García-Calero et al. 2020), which appears comparable to the primate inferior ganglionic eminence. Thus, in spite of its name suggesting a subpallial location, the inferior ganglionic eminence would be the progenitor zone of the amygdalar pallium. According to García-Calero et al. (2020), this amygdalar pallial sector contains at least four radial subunits giving rise to different neuron subgroups of the basal complex and cortical amygdala. However, the PL was not considered in this study, and its exact origin remains elusive. This information could help to better understand the topological location and possible migratory routes of immature cells found postnatally in the PL of different mammals. The aim of this study was to investigate the distribution of immature cells in the basolateral amygdalar complex of juvenile swine brains, trying to identify the PL as well as the phenotype and possible migratory routes of PL immature cells. To that aim, we performed immunolabeling for DCX, combined with the cell proliferation marker Ki-67 and different neuronal markers, including NeuN (neuronal nuclear protein, also known as FOX3), the transcription factors BRN2 (POU3F2, present in subsets of glutamatergic neurons) and COUP-TFII / NR2F2 (critical for amygdalar development and adult phenotype maintenance, and present in PL cells in both human and mouse). To distinguish PL from intercalated amygdalar cell clusters, we combined DCX with the transcription factor FOXP2. To better understand PL cytoarchitecture with respect to that of other amygdalar nuclei, we also performed Nissl staining in complete series of sections adjacent to those processed for immunohistochemistry of immunofluorescence. Our results demonstrated that the swine pallial amygdala shows a prolonged postnatal plasticity similar to that of humans and contains a reservoir of immature neurons coexpressing DCX and COUP-TFII / NR2F2 mainly located in the PL. We also identified the possible origin of these cells in a posteroventral ventricular/subventricular pallial sector adjacent to the temporal horn of the lateral ventricle expressing COUP-TFII / NR2F2.

## Materials and methods

In the present study, we used brains from swine *(Sus scrofa domesticus*) provided by the Applied Biomedical Research Centre (CREBA) of the Institute of Biomedical Research of Lleida- Dr. Pifarré Foundation (IRBLleida, Spain), located in Torrelameu (Lleida, Spain) (registered as a user center of experimental animals: L9900008; REGA: ES252310036907). In total, we used 4 juvenile swine of both sexes (1 male and 3 females), ranging in age from 2.5 to 3.5 months, and weighted between 30 and 40 kg. At this age, pigs are considered juvenile, as they have not yet reached sexual or full neurodevelopmental maturity. Animals were housed and handled in the pig CREBA facilities, according to the protocol approved by the Animal Experimentation Ethics Committee of the CREBA and following the regulations and laws of the European Union (Directive 2010/63/EU) and the Spanish Government (Royal Decrees 53/2013) for the care and handling of animals in research. These animals were previously used for practicing and improving surgical procedures by M.D. surgeons. After, animals were euthanized, and their brains were extracted and processed as explained below.

### Tissue collection and fixation

For cerebral tissue collection, the animals previously employed for surgery research were sacrificed, just before brain extraction, with a lethal dose of sodium pentobarbital (200 mg/kg; IV). After extracting the brain, the tissue was washed with abundant water to remove blood and hemispheres were separated and fixed by immersion in a solution of 4% paraformaldehyde (PFA) in 0.1 M phosphate buffer (PB), for 2–4 days.

### Sample preparation and sectioning

Some hemispheres were cut into four blocks (frontal, temporoparietal, occipital, and brainstem with cerebellum), while others were conserved in a single piece. Following fixation, the tissue was cryoprotected in a solution of increasing glycerol concentrations (10% and 20%) and 2% DMSO in 0.1 M PB for 6–10 days. The tissue was frozen by immersion in -65/-70 °C isopentane (2-methyl butane, Sigma-Aldrich, Germany) for 1–2 min, following the protocol of Rosene et al. (1986). Frozen brains were wrapped in aluminum foil and stored in a container at -80 °C until further use.

For sectioning, samples were cut using a freezing sliding microtome (Microm HM 450; Thermo Fisher Scientific), equipped with a large size Freezing Stage (BFS-40MPA, Physitemp Instruments, LLC, USA). Coronal, sagittal and horizontal sections of 100 μm thick were collected at 4 °C in 0.1 M PB, into twelve parallel series with about 20 sections per each block for coronal sections and each sagittal- or horizontally sectioned hemisphere. For each series, selected sections of the appropriate levels including the areas of interest were processed for studying. The sections not used immediately were stored in a 30% sucrose PB solution and frozen at -20 °C.

In one animal (male), the right hemisphere was sectioned in the sagittal plane, and two parallel series were stained for Nissl and immunohistochemistry for DCX. Similarly, in a female, the right hemisphere was sectioned in the horizontal plane, and two parallel series were processed for Nissl and DCX-immunohistochemistry staining. Horizontal sections from another female were processed, but in this case the occipital lobe of the right hemisphere was separated and stored for further analysis. Regarding frontal sections, the amygdala was studied from anterior to posterior levels (from 10.375 mm to 6.125 mm antero-posterior stereotaxic coordinates from the MRI Atlas, Saikali et al. 2010) by using Nissl and immunohistochemistry for DCX staining.

Following the initial analysis of Nissl and DCX chromogenic immunostained sections, selected frontal, sagittal and horizontal sections were processed for double and triple immunofluorescence, combining DCX with other markers of interest (see Table 1). Overall, 55 sections were Nissl-stained (10 frontal sections, 25 horizontal sections and 20 sagittal sections), about 100 sections were processed for single immunohistochemistry (including DCX, COUP-TFII, FOXP2 and Ki-67 staining), and 50 sections were processed for double or triple immunofluorescence.

### Nissl staining

To study the tissue organization and to help with the identification of the areas, complete series of sections of each plane were processed for Nissl staining as follows. After being mounted with a solution of gelatine from porcine skin at 0.5% in Tris buffer and dried, slides with sections were sequentially immersed in 100% ethanol and xylene to dissolve out the lipids and allow a better staining. After, sections were dried and incubated for 1 min with filtered 1% Toluidine blue in 0.1 M acetate buffer at pH 4.6. Sections were then rinsed with abundant water, and differentiated with acid alcohol (0.01% acetic acid solution in 70% ethanol) for 90 s. Subsequently, the slides were dehydrated with ascending ethanol concentrations (70%, 96%, and 100%), followed by a brief xylene wash, and finally coverslipped with Permount mounting medium (Thermo Fischer Scientific).

### Immunohistochemistry

Free-floating brain sections were processed for immunohistochemistry to detect DCX, COUP-TFII, FOXP2, or Ki-67, using specific primary antibodies (Table 1). Sections were washed with 0.1 M PB and then processed for antigen retrieval by incubation in a sodium citrate buffer (10mM Sodium Citrate, 0.05% Tween 20, pH 6.0) for 1 h at 60 °C. After rinsing, sections were permeabilized with PB containing 0.3% Triton-X 100 (PB-Tx; 0.1 M), and incubated for 2 h at room temperature in a blocking solution containing 2% of bovine serum albumin (BSA) and 20% of normal serum (according to the species in which the secondary antibody was raised) in 0.1 M PB. Next, sections were incubated in primary antibody solution (for dilutions see Table 1) in PB-Tx for 72 h at 4 °C with gentle shaking. After rinsing, sections were incubated in a biotinylated secondary antibody (Table 2) in PB-Tx overnight, at 4 °C with gentle shaking. Successively, sections were washed, and endogen peroxidase was blocked with a solution of PB with 20% methanol and 2% H_2_O_2_ for 20 min. After, sections were washed and incubated in the avidin–biotin complex (AB Complex, Vector Laboratories Ltd.) for 1 h at room temperature. After incubation in the ABC solution, the sections were first washed in PB, followed by Tris buffer (0.05 M, pH 7.6).

To reveal the staining, the sections were incubated with diaminobenzidine (DAB), prepared according to the manufacturer’s instructions (SIGMAFAST™ tablets, Sigma-Aldrich Co. LLC; or DAB Chromogen/Substrate Bulk Pack, ScyTek Laboratories INC.). The reaction was stopped by rinsing the sections with Tris buffer. Sections were then mounted, dehydrated and covered with Permount™ as described before.

As negative control, some sections were processed following the immunohistochemistry procedure but omitting primary antibodies. No specific cell labeling was observed (Freixes et al. 2025).

**Table 1:** Primary antibodies

Type	Antibody	Antigen recognized	Dilution	Manufacturer and reference	RRID
Polyclonal	Goat anti-doublecortin	Doublecortin C-18	1:1000–1:2000 for IHC/IF 1:1000 for WB	Santa Cruz Biotechnologies Ref. sc-8066	AB_2088494
	Rabbit anti-doublecortin	Doublecortin	1:3000 for IHC/IF 1:1000 for WB	Abcam Ref. ab18723	AB_732011
	Rabbit anti-FOXP2	Forkhead box P2	1:2000 for IHC/IF	Abcam Ref. ab16046	AB_2107107
	Rabbit anti-actin	N-terminal actin peptide attached to a multiple antigen peptide backbone.	1:5000 for WB	Sigma-Aldrich Ref. A5060	AB_476738
Monoclonal	Rat anti-Ki-67	Ki-67 SolA15	1:100 for IHC/IF	Invitrogen Ref. 14-5698-82	AB_10854564
	Mouse anti-NeuN	Neuronal Nuclei (NeuN); clone A60	1:1000 for IHC/IF	Sigma-Aldrich Ref. MAB377	AB_2298772
	Mouse anti-COUP-TF2/NR2F2	COUP-TF II/NR2F2 clone H7147	1:500 for IHC/IF 1:1000 for WB	Bio-Techne R&D Systems Ref. PP-H7147-00	AB_1964214
	Mouse anti-BRN2 IgG1	POU class 3 homeobox 2	1:200 for IF	Santa Cruz Biotechnologies Ref. sc-393,324	AB_2737347
	Mouse anti-actin	Slightly modified β-cytoplasmic actin N-terminal peptide.	1:5000 for WB	Sigma-Aldrich Ref. A5441	AB_476744

### Double and triple immunofluorescence

Multiple labeling immunofluorescence was performed to detect cells coexpressing different markers. Following tissue permeabilization and blocking of non-specific binding, sections were incubated at 4 °C for 72 h with a cocktail of primary antibodies (Table 1). After rinsing, sections were incubated overnight at 4 °C in PB-Tx with the corresponding cocktail of fluorescent secondary antibodies (Table 2). Finally, the sections were mounted with a solution of gelatine at 0.5% and covered with antifading mounting medium (Vectashield Hardset Antifade mounting medium, Vector Laboratories Ltd.).

As done for immunohistochemistry, some sections were incubated omitting the cocktail of primary antibodies to ensure their specificity. No labelling was detected.

**Table 2:** Secondary antibodies

Type	Antibody	Dilution	Manufacturer and reference	RRID
Biotinylated for IHC	Rabbit anti-goat IgG (H + L)	1:200	Vector Laboratories Ref. BA-5000	AB_2336126
	Goat anti-mouse IgG (H + L)	1:200	Vector Laboratories Ref. BA-9200	AB_2336171
	Goat anti-rabbit IgG (H + L)	1:200	Vector Laboratories Ref. BA-1000	AB_2313606
	Goat Anti-Rat IgG (H + L)	1:200	Vector Laboratories Ref. BA-9400	AB_2336202
HRP-linked for WB	Anti-rabbit IgG	1:20000	Cell Signaling Ref. 7074	AB_2099233
	Anti-mouse IgG	1:20000	Cell Signaling Ref. 7076	AB_330924
	Anti-goat IgG	1:5000	Vector Laboratories Ref. PI-9500	AB_2336124
Fluorescent for IF	Donkey anti-goat IgG (H + L), coupled to Alexa 488	1:500	Jackson ImmunoResearch Ref. 705-546-147	AB_2340430
	Donkey anti-mouse IgG (H + L), coupled to Alexa 488	1:500	Invitrogen Ref. A21202	AB_141607
	Donkey anti-goat IgG (H + L), coupled to Alexa 568	1:500	Invitrogen Ref. A11057	AB_2534104
	Donkey anti-rabbit IgG (H + L), coupled to Alexa 568	1:500	Invitrogen Ref. A10042	AB_2534017
	Goat anti-rabbit IgG (H + L), coupled to Alexa Plus 405	1:250	Invitrogen Ref. A48254	AB_2890548
	Goat anti-mouse IgG (H + L), coupled to Alexa Plus 405	1:250	Invitrogen Ref. A48255	AB_2890536
	Goat anti-rat IgG (H + L), coupled to Alexa Plus 405	1:250	Invitrogen Ref. A48261	AB_2890550

### Western blotting for validation of primary antibodies

Since the employed antibodies were not validated by the respective manufacturers for swine tissue, we employed the Western blot technique for validation. Samples consisted of brain tissue, obtained from juvenile and embryos at 50 days of gestation (E50).

One hemisphere of a juvenile swine was dissected to obtain samples from the caudate nucleus (Cn), the piriform cortex (Pir) and the neocortex (NCx), while for the embryonic sample, one whole hemisphere was used. All the samples were fragmented and homogenized using an electric homogenizer (Tissue Grinder) in ice-cold radioimmunoprecipitation assay (RIPA) containing lysis buffer (150 mM NaCl; 1% NP-40; 0.5% Na-deoxycholate; 0.1% SDS; 50 mM Tris-HCl [pH 7.4]), protease inhibitor (Sigma-Aldrich, cat # P8340) and PhosSTOP (Roche). Homogenized samples were centrifuged at 12,000 rpm for 15 min at 4 °C and then protein concentrations of the supernatants were determined by BIO-RAD Micro DC protein assay (BIO-RAD, Laboratories, Inc.). Samples containing 20–40 µg of protein were mixed with an equivalent volume of sample buffer containing 8% of SDS and 2% mecaptoethanol and heated at 100 °C for 5 min. Following, samples were first loaded onto a denaturing 10% sodium dodecyl sulphate-polyacrylamide gel, together with chemiluminescent (MagicMark™ XP, Invitrogen) and multicolor prestained (PageRuler™ Plus, Thermo Fisher) ladders. Proteins were separated by electrophoresis for 90 to 120 min at an intensity of 25 mA per gel and then were electrotransferred to a nitrocellulose membrane in Tris-glycine-methanol buffer, using semidry transfer. The membrane was blocked for 1 h at room temperature in a blocking solution mixture of 5% nonfat dry milk in 0.1% Tween 20 and Tris-buffered saline pH 8 (TBST). After washing with TBST, membranes were incubated overnight at 4 °C with anti-DCX and anti-COUP-TFII antibodies (Table 1) for immunodetection. Next day, membranes were washed and incubated with the appropriate peroxidase-conjugated secondary antibodies (Table 2) for 60 min at room temperature, washed in TBST, and visualized using the ECL Prime Western blotting Detection Reagent detection kit (GE Healthcare), as described by the manufacturer. Chemi-Doc MP Imaging System (BIO-RAD Laboratories, Inc.) was used for the visualization of bands. Following, the membrane was incubated with anti-actin antibody as loading controls.

Doublecortin antibodies showed a band of 40 kDa, as described by the manufacturer (Supplementary Fig. 1). Similarly, brain sample reacted with COUP-TFII antibody giving a band of a similar weight as the one reported by the manufacturer (~ 55 kDa).

**Fig. 1:**
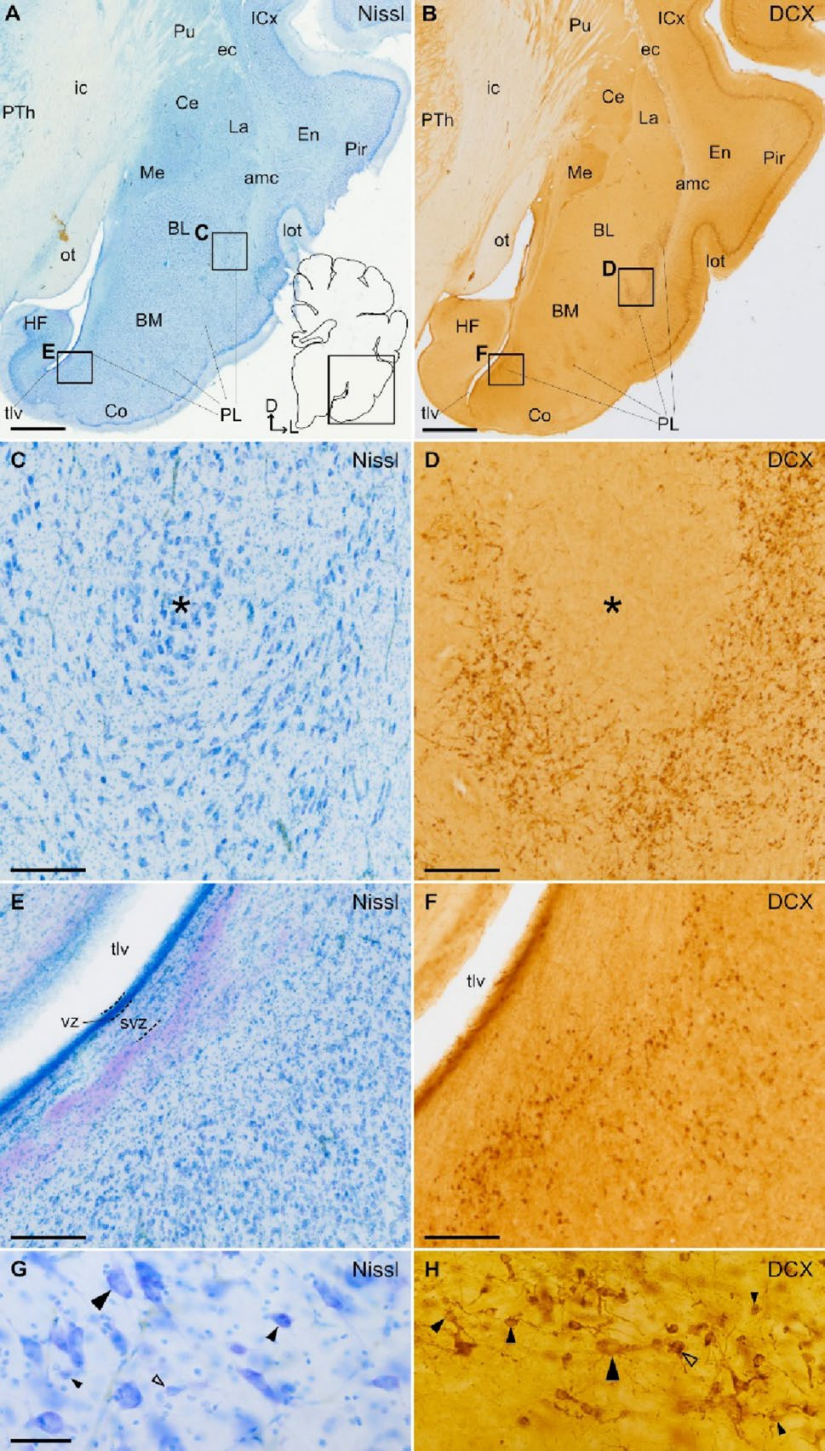
DCX expression in the paralaminar nucleus of the juvenile swine pallial amygdala. **A**, **B** Parallel frontal sections at a similar level of the juvenile swine amygdala stained for Nissl (**A**) and immunohistochemistry for DCX (**B**). Note the DCX+ cell clusters near the external and ventromedial borders of the basal amygdalar complex, within the paralaminar nucleus (PL). Squared areas in A are shown at higher magnification in C and E, while squared areas in B are shown at higher magnification in D and F. **C**, **D** Details of lateral subdivision of PL showing shell-like clusters of DCX+ cells with small, round or fusiform somas, that surround islands of non-immunoreactive, large cells (islands are pointed with asterisks). **E**, **F** Details of medial subdivision of PL containing groups of small DCX+ cells near the ventricular (vz) and subventricular (svz) zones, adjacent to the temporal horn of the lateral ventricle (tlv). In contrast to those of lateral PL, DCX+ cells of medial PL do not display a shell-like organization. **G**, **H** Details showing different morphologies in the lateral subdivision of PL. **G** With Nissl staining, large cells (large filled arrowhead) are mixed with medium-sized and small round cells (medium and small filled arrowheads). Some of the latter are seen in pairs with the somas apposed (empty arrowhead). **H** With DCX immunohistochemistry, the majority of cells have small or medium-sized rounded somas (small and medium filled arrowheads), and some of them were in pairs showing perikaryal apposition (empty arrowhead). We also observed other cells with large soma and pyramidal-like morphology, some of which showed light DCX immunoreactivity suggesting a higher degree of maturation (empty arrowhead). A schematic representation of the frontal section, and the mediolateral and dorsoventral axes are shown in A for orientation. For other abbreviations, see list. Scales: A, B = 2 mm; C, D, E, F = 200 μm; G, H = 50 μm

### Identification of regions of interest

To identify the regions of interest, we used our own Nissl-stained sections and those of the wild boar brain from the University of Wisconsin-Madison, as well as the MRI 3D atlas from Saikali et al. (2010). Our identification was based on both the topological position and cytoarchitecture of the structures.

### Digital photographs and figures

Digital microphotographs of the sections processed for immunohistochemistry were taken on a Leica microscope (DM2500 LED, Leica Microsystems GmbH) equipped with a digital camera (Zeiss Axiovision Digital Camera -Carl Zeiss- and Flexacam C5 LSR Camera -Leica Microsystems GmbH-). Serial images from fluorescent material were taken under confocal microscopes (Olympus FV1000 -Olympus Corporation- and Nikon AX NSPAR -Nikon). Selected digital fluorescent images were adjusted and extracted using Olympus FV10-ASW 4.2 Viewer (Olympus Corporation) and Image J (Fiji). Finally, the figures were mounted using Affinity Designer 2 (version 2.6.5, Serif Corporation).

## Results

We analyzed coronal, sagittal, and horizontal sections from juvenile swine brains of both sexes, at different levels, to carry out a systematic description of the distribution of DCX+ cells throughout the amygdala. The expression pattern of DCX was similar across animals and in both sexes, even if the limited brain number did not allow a reliable comparison between sexes or ages. We found DCX+ cells in the basal complex of the amygdala, mainly localized in clusters or patches along the external border of the complex, resembling in topological position the PL. To know more about the phenotype of the DCX+ cells of the clusters and distinguish whether they truly represent the swine PL or belong to clusters of intercalated amygdalar cells (ITC), we compared the results between adjacent sections single or double/triple-labeled for DCX, NeuN (that is expressed in neurons since they start to differentiate), and/or the transcription factors BRN2 (POU3F2, which is expressed in subpopulations of immature glutamatergic cortical neurons), COUP-TFII (NR2F2, which is crucial for amygdala patterning and is expressed in PL neurons), and FOXP2 (that is expressed in intercalated cells of the amygdala). As many of the DCX + cells in the clusters had a migratory cell morphology and some of them formed chains (in agreement with Torrijos- Saiz et al. 2025; Freixes et al. 2025), we tried to identify possible progenitor areas and explore the possible migratory pathways that immature cells might follow from these progenitor areas to their final position in PL. To help in this analysis, we also studied the coexpression of Ki-67, a mitotic marker, with other markers.

### Distribution of DCX+ cells in the pallial amygdala and identification of swine PL

**Fig. 2:**
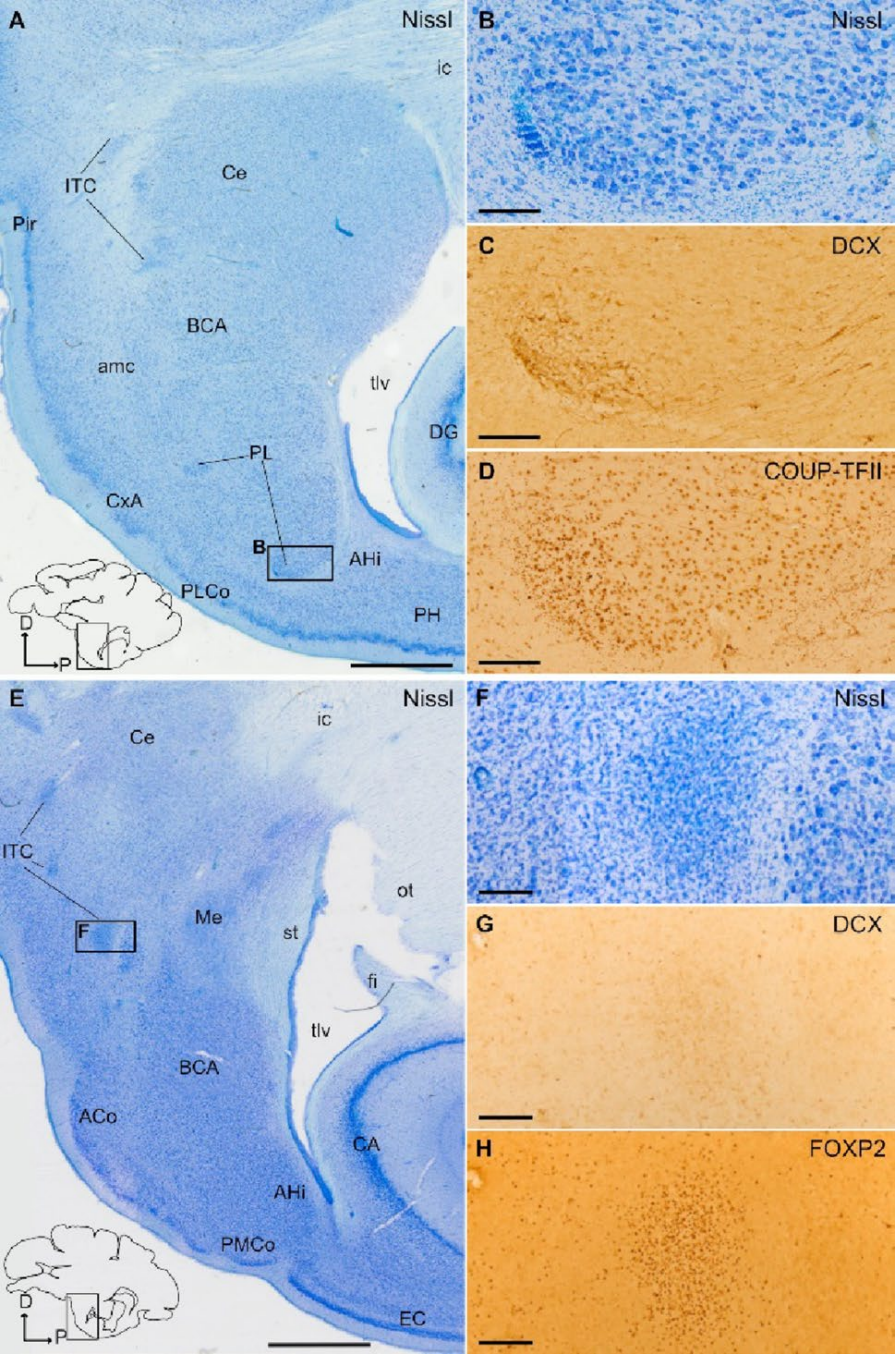
DCX and COUP-TFII expression in the paralaminar nucleus (PL), and distinction from the intercalated amygdalar cells. **A**–**D** Parallel sagittal sections or details at similar level of the amygdala stained for Nissl (**A**, detail in **B**) and immunohistochemistry for DCX (**C**) or COUP-TFII (**D**). Squared area in A is shown at higher magnification in B. **B**–**D** Details of a DCX+ cell cluster in the lateral subdivision of PL, showing a high density of small cells, adjacent to the amygdalar capsule (amc) (**B**, **C**). Abundant COUP-TFII immunoreactive cells with small, round or fusiform somas are also observed in lateral PL, overlapping the DCX+ cells (**D**). In addition, many COUP-TFII+ large cells are found in PL as well as in other nuclei of the basal amygdalar complex (BCA). **E**–**H** Parallel sagittal sections or details at a similar level of the amygdala, medial to the sections shown in (A-D), stained for Nissl (E, detail in F), or processed for immunohistochemistry for DCX (G) or FOXP2 (**H**). Squared area in E is shown at higher magnification in F. **F**–**H** Details of a cluster of intercalated amygdalar cells (ITC) rich in FOXP2 immunoreactivity (**H**). In contrast to PL cell clusters, ITC clusters do not express or only show negligible immunoreactivity for DCX (**G**). Schematic representations of the sagittal sections, and the anteroposterior and dorsoventral axes are shown in A and E for orientation. For other abbreviations, see list. Scales: A, E = 2 mm; B-D, F-H = 200 μm

**Fig. 3:**
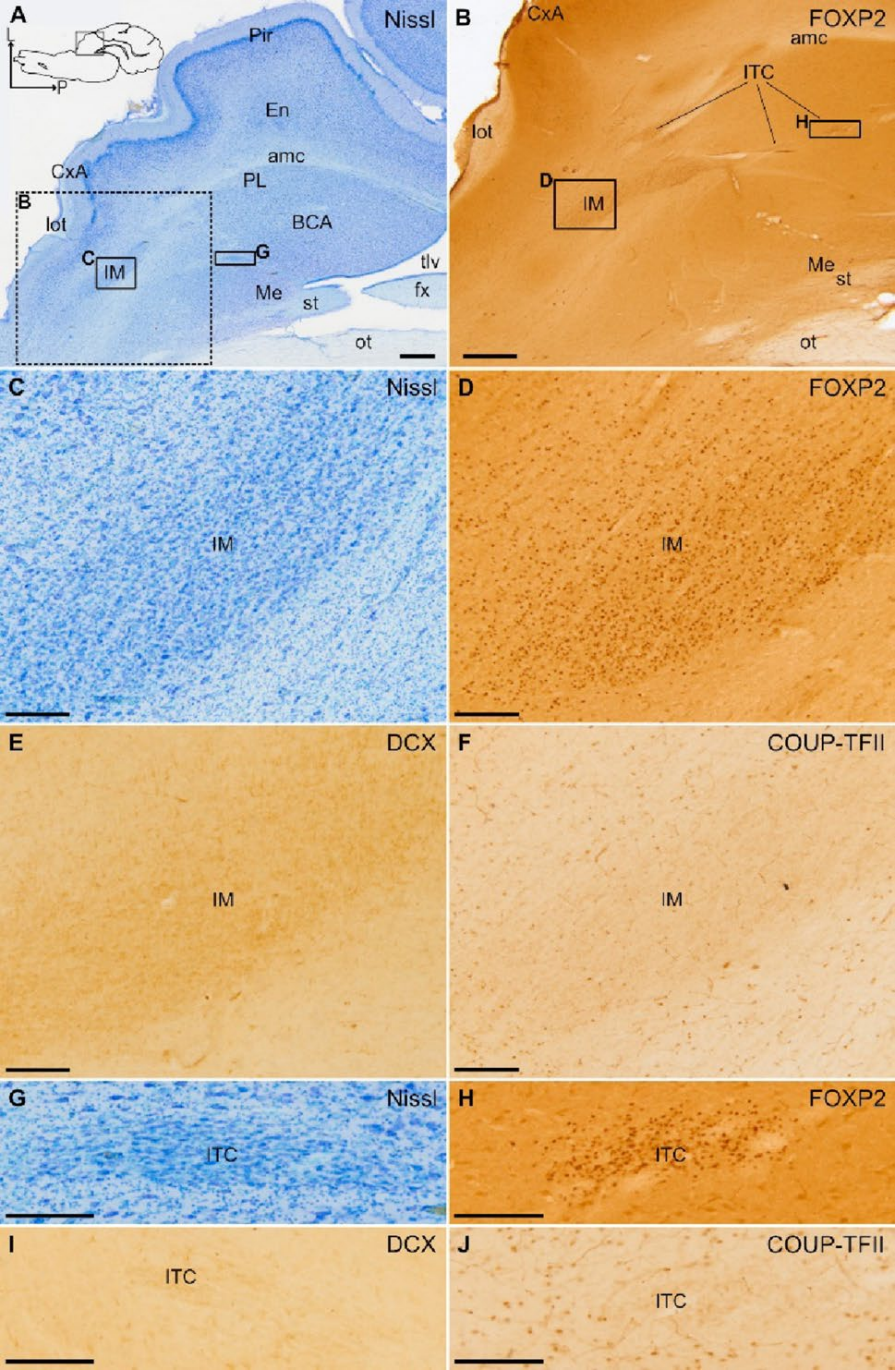
Identification of the intercalated amygdalar cells (ITC) of juvenile swine. **A**–**J** Parallel horizontal sections at a similar level of the amygdala stained for Nissl (**A**, details in **C** and **G**) and immunohistochemically stained for FOXP2 (**B**, details in **D** and **H**), DCX (**E**, **I**) or COUP-TFII (**F**, **J**). Dashed square in A is shown at higher magnification and immunohistochemically stained for FOXP2 in B. Squared areas in A are shown at higher magnification in C and G. Squared areas in B are shown at higher magnification in D and H. Note the abundance of FOXP2 immunoreactive cells in ITC, including its main nucleus (IM). **C**–**F** Details of IM showing a high density of cells, immunoreactive for FOXP2 (D), but with negligible expression of DCX (**E**) and no expression of COUP-TFII (F). Similarly, **G**–**J** show an ITC cluster with high density of FOXP2 + cells (H), but with negligible expression of DCX (I) and no expression of COUP-TFII (J). This is a major difference of the ITC clusters with those of the paralaminar nucleus (seen in previous figures). In addition, ITC clusters are often located anterior or medial to the basal amygdalar complex (BCA), while the PL is mainly located along the external and ventromedial margins on BCA. A schematic representation of the horizontal section, and the anteroposterior and mediolateral axes are shown in A for orientation. For other abbreviations, see list. Scales: A, B = 1 mm; C-J = 200 μm

The pallial amygdala consists of the basal complex of the amygdala (BCA) and several cortical amygdalar areas near the surface. Like in other gyrencephalic mammals, the swine pallial amygdala has a relatively large size, as compared to the central and medial amygdalar nuclei (Fig. 1A and Supplementary Fig. 2). We found numerous DCX+ cells in both the BCA and the cortical areas of juvenile swine, indicating high levels of plasticity. The majority of the DCX+ cells were observed near the external border of BCA, close to the amygdalar capsule, and in its posteroventral continuation towards the subventricular zone adjacent to the temporal horn of the lateral ventricle (Figs. 1 and 2). These cells resembled the primate PL in topological position and postnatal DCX expression. Like primate PL, DCX+ cells of swine PL were often broken into clusters which overlapped with COUP-TFII expressing cells (Fig. 2C, D), but not with FOXP2, which identified the swine intercalated amygdalar cells (Figs. 2F-H and 3B, D and H). In contrast to PL clusters, the intercalated cell clusters showed negligible levels of DCX immunoreaction (Figs. 2G and 3E and I) and did not express COUP-TFII (Fig. 3F, J). The intercalated cell clusters and those of PL also differed in location: while the first were mainly found anterior to BCA (the main intercalated nucleus or IM) or along its medial border with the central or medial amygdala (Fig. 3), the PL clusters were mainly observed laterally and became more abundant at posterior levels (Figs. 2 and 3 and Supplementary Fig. 2).

Like in primates, the swine PL also displayed lateral and medial subdivisions that surrounded laterally and ventromedially the BCA (Fig. 1B; details of lateral and medial subdivisions are shown in Fig. 1D and F, respectively). DCX+ cells of the lateral subdivision were organized in patches or clusters, which were more abundant ventrally, adjacent to the basolateral (BL) and basomedial (BM) nuclei (Fig. 1B, Supplementary Fig. 2). In frontal sections, clusters of DCX+ cells of the lateral PL displayed a shell-like organization, surrounding islands of non-stained large cells of the BCA (Fig. 1C, D). As seen in Nissl staining, the PL cells of the shell-like clusters showed different soma sizes and shapes, including large fusiform cells (Fig. 1C), large oval cells (large arrowhead in Fig. 1G), and medium-sized or small round cells f (medium and small arrowheads in Fig. 1G). Some of these different cell morphologies were also seen with DCX immunohistochemical labeling, with a predominance of small, round cells (filled arrowheads of small or medium sizes in Fig. 1H). We also observed DCX+ cells with large pyramidal-like morphology (large, filled arrowhead). The light immunoreactivity in some of the latter cells suggested that they have a higher degree of maturity (large, filled arrowhead in Fig. 1H). In the medial PL, DCX+ cells were more dispersed and rarely formed clusters, although these did not display a shell-like organization (Fig. 1E, F). Different morphologies and soma sizes were observed in medial PL but most DCX+ cells were round and small (Fig. 1F).

### Phenotype of DCX+ cells in PL

**Fig. 4:**
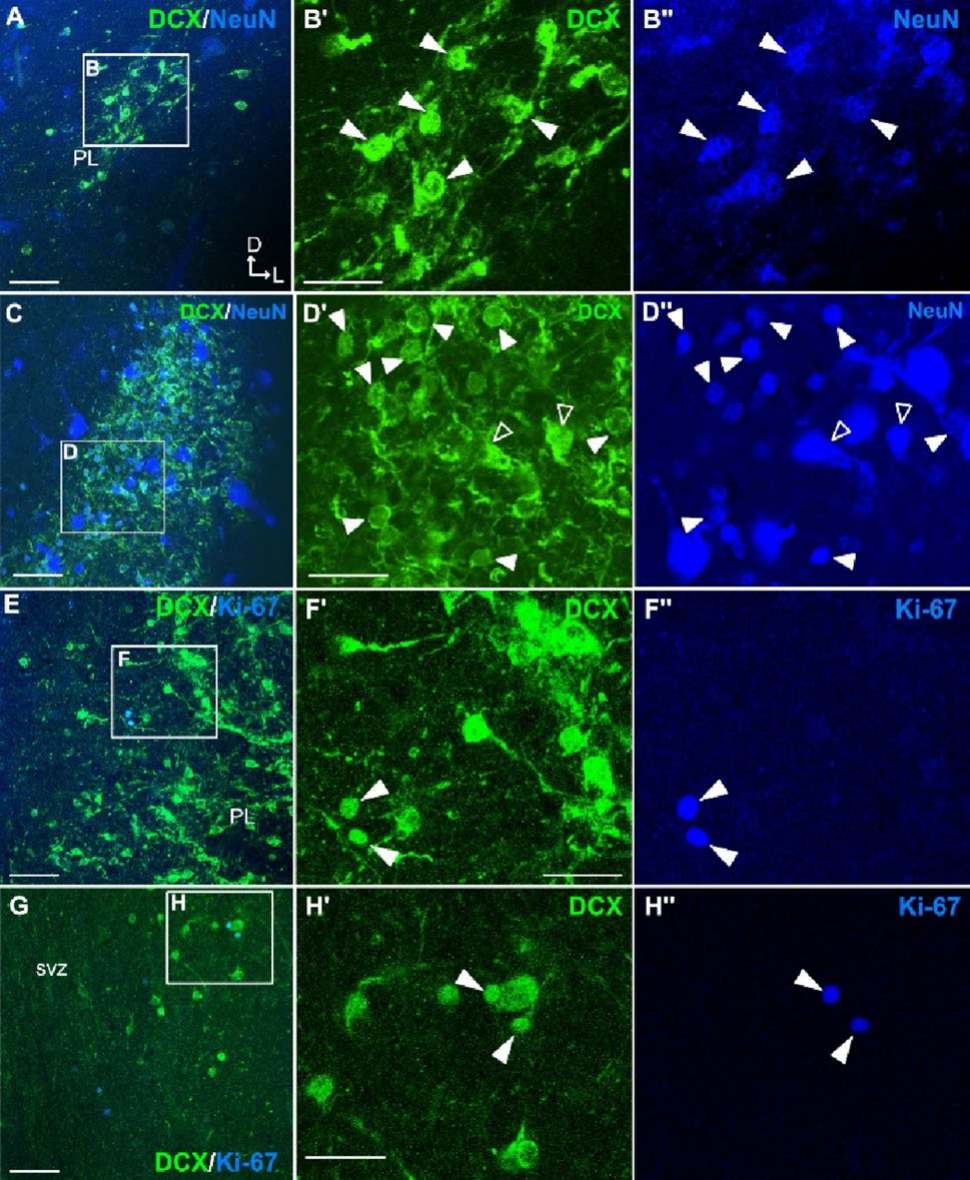
NeuN and Ki-67 expression in DCX+ cells of the swine paralaminar nucleus (PL). **A**–**D’’** Confocal images of a frontal section at the level of the lateral paralaminar nucleus (PL) (similar level to that Fig. 1), processed for immunofluorescence for DCX (green) and NeuN (blue). (**B’**-**B’’**, **D**’-**D’’**) Details of the squared areas in A and C, with separate confocal channels. These details show numerous coexpressing cells (arrowheads) in the lateral PL. Note that coexpressing cells are small and often have a round soma (filled arrowheads), while some others have a medium-sized soma and dendrites (empty arrowheads), but large NeuN-expressing cells do not coexpress DCX. **E**-**F’’** Confocal images of a frontal section (similar level to that Fig. 1) at the level of lateral PL (**E**-**F’’**) and medial PL (**G**-**H’’**), processed for immunofluorescence for DCX (green) and Ki-67 (blue). **F**-**F’’**, **H**-**H’’** Details of the squared areas in F and H, showing separate confocal channels. Note the scarce double labeled small cells in lateral and medial PL (arrowheads). Anteroposterior and mediolateral axes are indicated in A for orientation (applies to C, E and G). For other abbreviations, see list. Scales: A, C, E, G = 50 μm; B’,D’,F’,H’ = 25 μm (applies to B-B’’, D’-D’’, F’-F’’, H’-H’’)

**Fig. 5:**
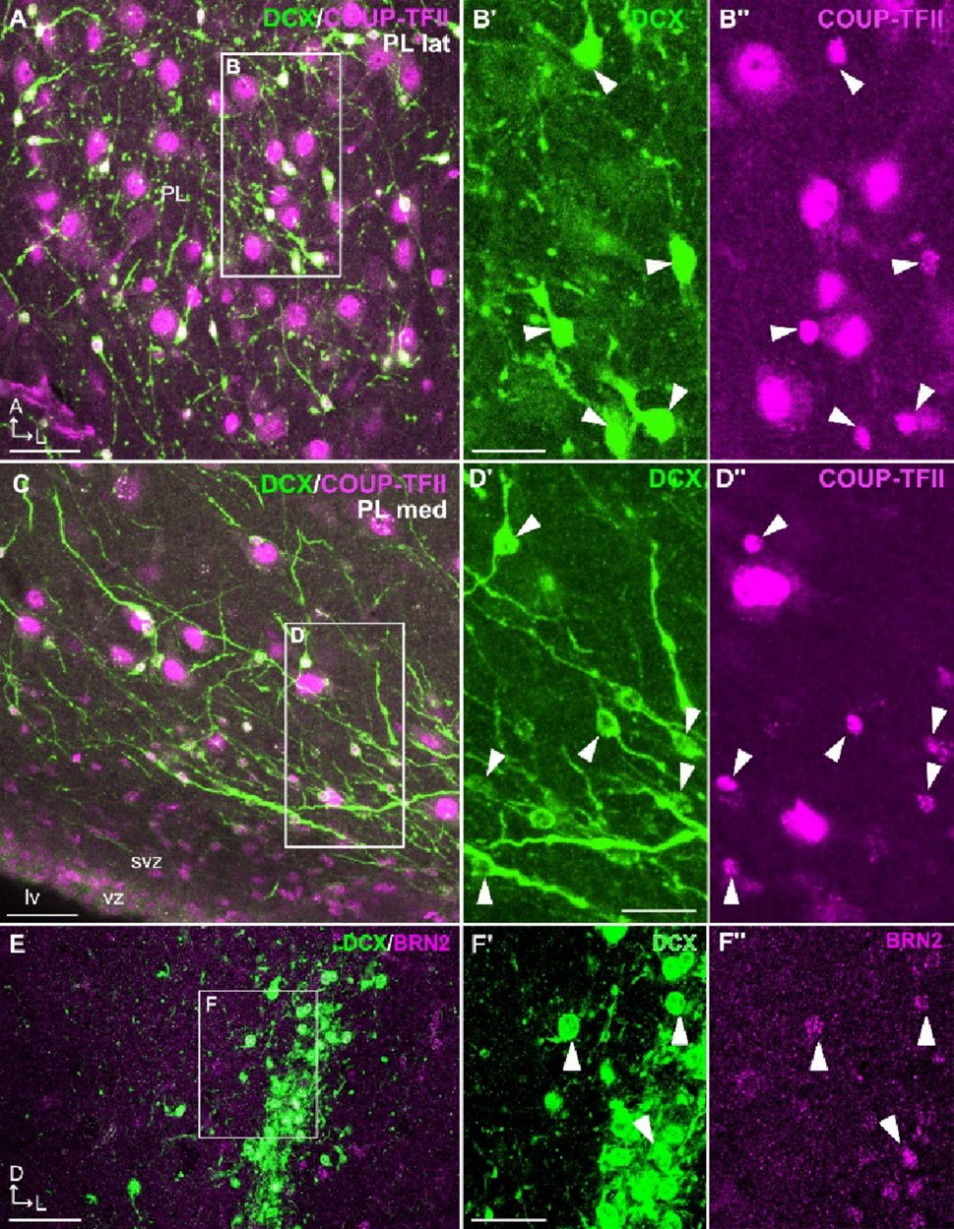
COUP-TFII and BRN2 expression in DCX+ cells of the swine paralaminar nucleus (PL). **A**-**D’’** Confocal images of a horizontal section (similar level to that in Fig. 3), at the level of the lateral PL (**A**-**B’’**) or medial PL (**C**-**D’’**), processed for immunofluorescence for DCX (green) and COUP-TFII (magenta). (**B’**-**B’’**, **D’**-**D’’**) Details of the squared areas in A and C, showing separate confocal channels. Arrowheads point to numerous double labeled cells, which usually display small, round or fusiform somas. Note that the large COUP-TFII immunoreactive cells do not coexpress DCX. **E**-**F’’** Confocal images of a frontal section (similar level to that in Fig. 1) processed for immunofluorescence for DCX (green) and BRN2 (blue). **F’**, **F’’** Details of the squared area in E, showing separate confocal channels. Note the presence of a few double-labeled cells (arrowheads). Anteroposterior and mediolateral axes are indicated in A for orientation (also applies to C). Dorsoventral and mediolateral axes are indicated in E for orientation. For other abbreviations, see list. Scales: A, C, E = 50 μm; B’-B’’ = 25 μm (also applies to D’-D’’, F’-F’’)

**Fig. 6:**
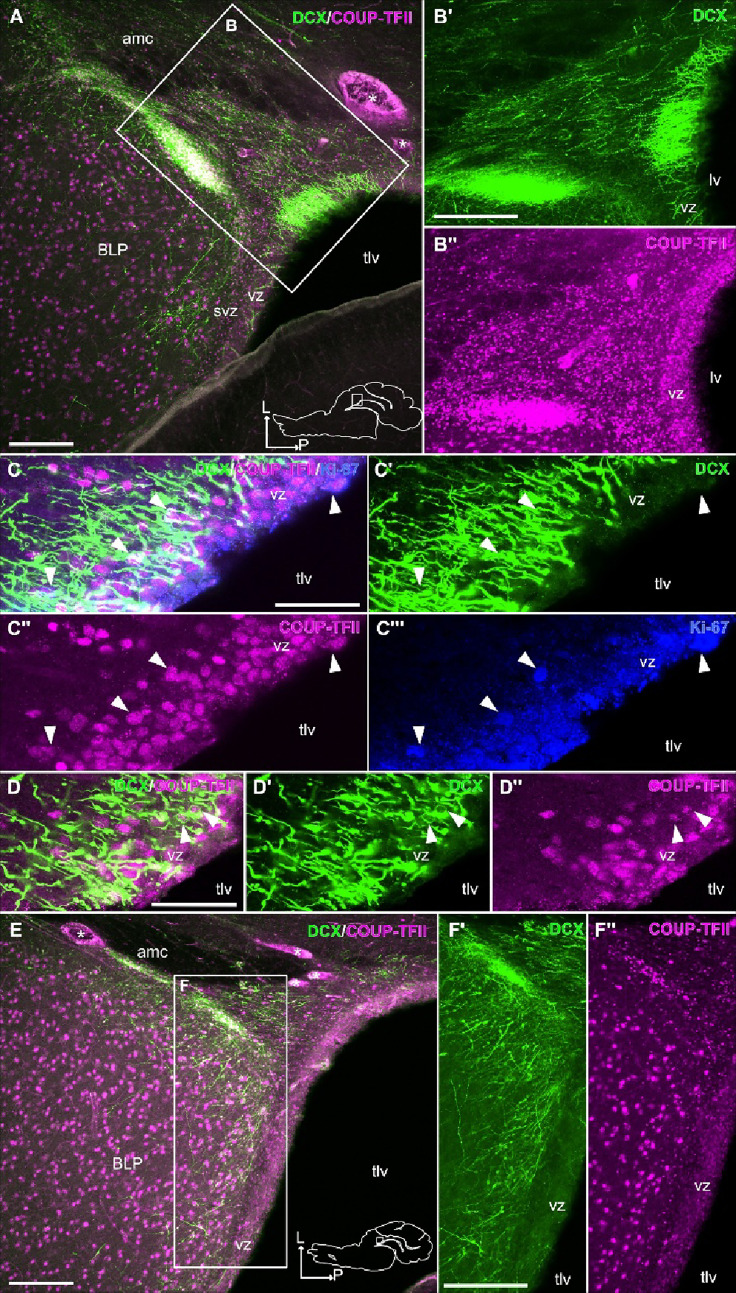
DCX+, COUP-TFII + and Ki-67 + cells in the posterolateral inferior ventricular and subventricular zones (vz/svz), adjacent to the temporal horn of the lateral ventricle (tlv). **A**–**D’’** Confocal images of a horizontal section processed for immunofluorescence for DCX (green), COUP-TFII (magenta) and Ki-67 (blue). **B’**-**B’’** Details at higher magnification of the squared area in A, with separate green and magenta confocal channels, showing abundant DCX immunoreactive cells and their radial processes (**B’**) and abundant COUP-TFII+ cells with small, round somas (**B’’**) in the posterolateral inferior part of the svz and vz. Note their continuity with labeled migratory-like cells in the inner part of the amygdalar capsule (amc) and a cluster of cells in the adjacent lateral paralaminar nucleus (PL). (**C**-**C’’’**) Detail of the vz/svz, showing DCX+ radial processes and some somas (**C’**; most cell somas are found in a different z plane), COUP-TFII+ small cells (**C’’**; immunoreaction is mainly found in the cellular nucleus) and abundant Ki-67 + cells (**C’’’**). Arrowheads point to COUP-TFII and Ki-67 double labeled cells. **D**-**D’’** Another detail of the vz/svz, showing the presence of some somas coexpressing DCX (**D**’) and COUP-TFII (**D’’**). **E**–**F’** Confocal images of a horizontal section (similar level to that in Fig. 3) processed for immunofluorescence for DCX (green) and COUP-TFII (magenta). **F’**-**F’’** Details at higher magnification of the squared area in E, with separate confocal channels, showing tangentially-oriented DCX immunoreactive cells and their processes, forming chains parallel or oblique to the ventricle. These cellular chains seem to separate from the posterolateral svz and extend toward the medial subdivision of PL (**F’**). These tangentially-oriented migratory-like cells also coexpress COUP-TFII (**F’’**). Schematic representation of the horizontal sections and anteroposterior and mediolateral axes are shown in A and E for orientation. Asterisks (_*_) in A and E indicate blood vessels. For other abbreviations, see list. Scales: A, B’, E, F’ = 250 μm (also applies to B’’, F’’); C, D = 50 μm (also applies to C’-C’’’, D’-D’’)

**Fig. 7:**
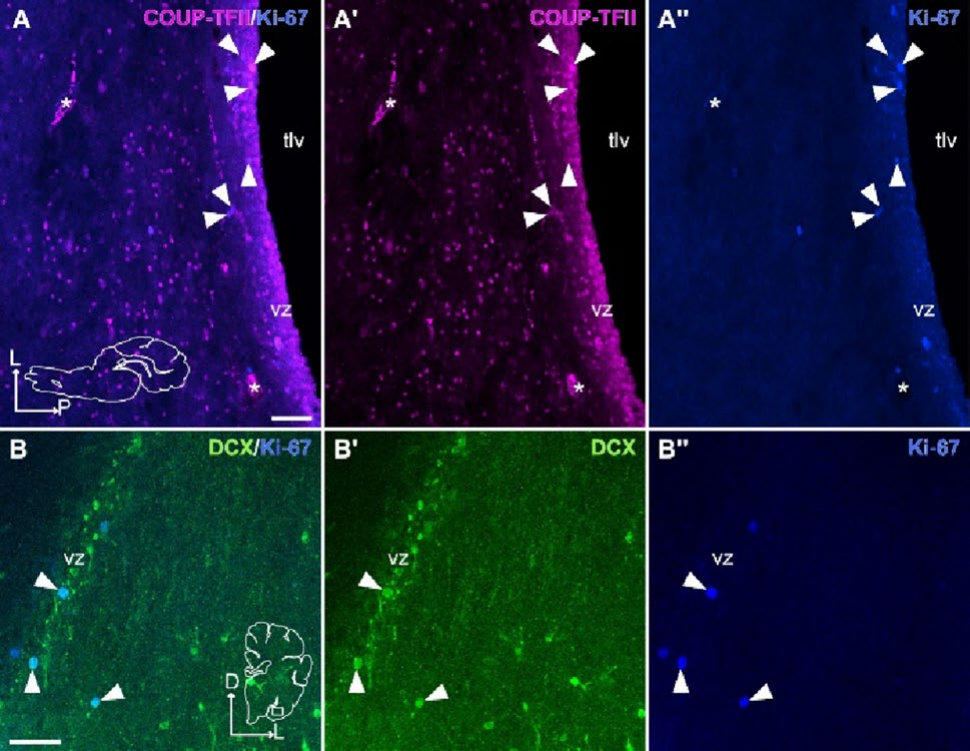
Ki-67+/COUP-TFII + and Ki-67+/DCX+ proliferating cells in the ventricular/subventricular zones (vz/svz). **A**-**A’’** Confocal images of a horizontal section (similar level to that in Fig. 3), at the level of the posterolateral inferior vz/svz, processed for immunofluorescence for COUP-TFII (magenta) and Ki-67 (blue). Detail of the vz/svz, with some cells coexpressing COUP-TFII (**A’**) and Ki-67 (**A’’**) (arrowheads point to double labeled cells). **B**-**B’’** Confocal images of a frontal section (similar level to that in Fig. 1), at the level of the medial PL showing immunofluorescence for DCX (green) and Ki-67 (blue) in the vz/svz. Some cells are coexpressing DCX (B’) and Ki-67 (**B’’**) (arrowheads). Schematic representations of the horizontal (**A**) and frontal (**B**) sections and the corresponding axes are indicated for orientation. For other abbreviations, see list. Scales: A, B = 50 μm (also applies to A’-**A’’**, **B’**-**B’’**)

To better understand the phenotype of the immature DCX+ cells of PL, we carried out double and triple immunofluorescence (Figs. 4, 5, 6 and 7). This combination also allowed a better correlation between distinct morphologies and soma sizes of DCX+ cells and specific phenotypes. The majority of the DCX+ cells of PL expressed the neuronal marker NeuN (Fig. 4A-D’’), and cells coexpressing DCX and NeuN (arrowheads in Fig. 4B’-B’’, D’-D’’) were generally smaller than those expressing only NeuN. Among the cells coexpressing DCX and NeuN we could distinguish at least two types, one with a larger (medium-sized) soma and dendrites (empty arrowheads in Fig. 4D’-D’’), and another one with very small and round or fusiform soma (filled arrowheads in Fig. 4A-D’’). Double labeling with Ki-67 showed that a few of the small and round DCX+ cells coexpressed Ki-67 (Figs. 4E-H’’), indicating that at least a few of these immature cells were proliferating in both lateral (Fig. 4E-F’’) and medial (Fig. 4G-H’’) parts of PL.

In addition to NeuN, most DCX+ cells in PL showed COUP-TFII immunofluorescence (Figs. 5A-D’’). As typical of transcription factors, COUP-TFII was mainly located in the cellular nucleus. However, we found COUP-TFII+ cell nuclei of two sizes, large and small. Almost all DCX+ cells showed a small COUP-TFII+ nucleus. Most DCX+ cells had a round or fusiform soma, some with a migratory-like morphology. Large COUP-TFII non-DCX cells were abundant in both PL and the rest of the BCA, and resembled mature neurons.

We only found very few DCX+ cells coexpressing BRN2 in PL (Fig. 5E-F’’), which is a major difference with our previous findings in the olfactory areas, where many cells coexpressed DCX and BRN2 (Freixes et al. 2025).

### Possible progenitor areas of DCX+ cells and migratory routes to PL

Many DCX+ cells in PL had an elongated morphology, and some displayed a leading process resembling migratory cells (Figs. 5A, B’,C, D’). Since the vast majority of the DCX+ cells of PL coexpressed COUP-TFII, we analyzed expression of this transcription factor in the ventricular/subventricular zones (vz/svz) of the temporal horn of the lateral ventricle (tlv) in order to understand the origin of PL immature cells. We identified a posterolateral inferior sector of the vz/svz rich in radially oriented DCX+ cells and small round COUP-TFII cells (Fig. 6). Remarkably, many cells in this vz/svz sector coexpressed COUP-TFII and Ki-67 and DCX and Ki-67 (Fig. 6C-C’’’, 7 A-A’’), resembling a hotspot for production of new cells. We also found coexpression of DCX and COUP-TFII (Fig. 6D-D’’). A major difference between this vz/svz sector and the Arc (giving rise to immature neurons of the rostral migratory stream and those found in the external capsule and many olfactory areas) is that the Arc did not express COUP-TFII (Supplementary Fig. 3). These observations discarded the Arc as a possible origin of immature cells found in PL and pointed to the posterolateral and inferior vz/svz sector adjacent to the tlv as the most likely origin.

**Fig. 8:**
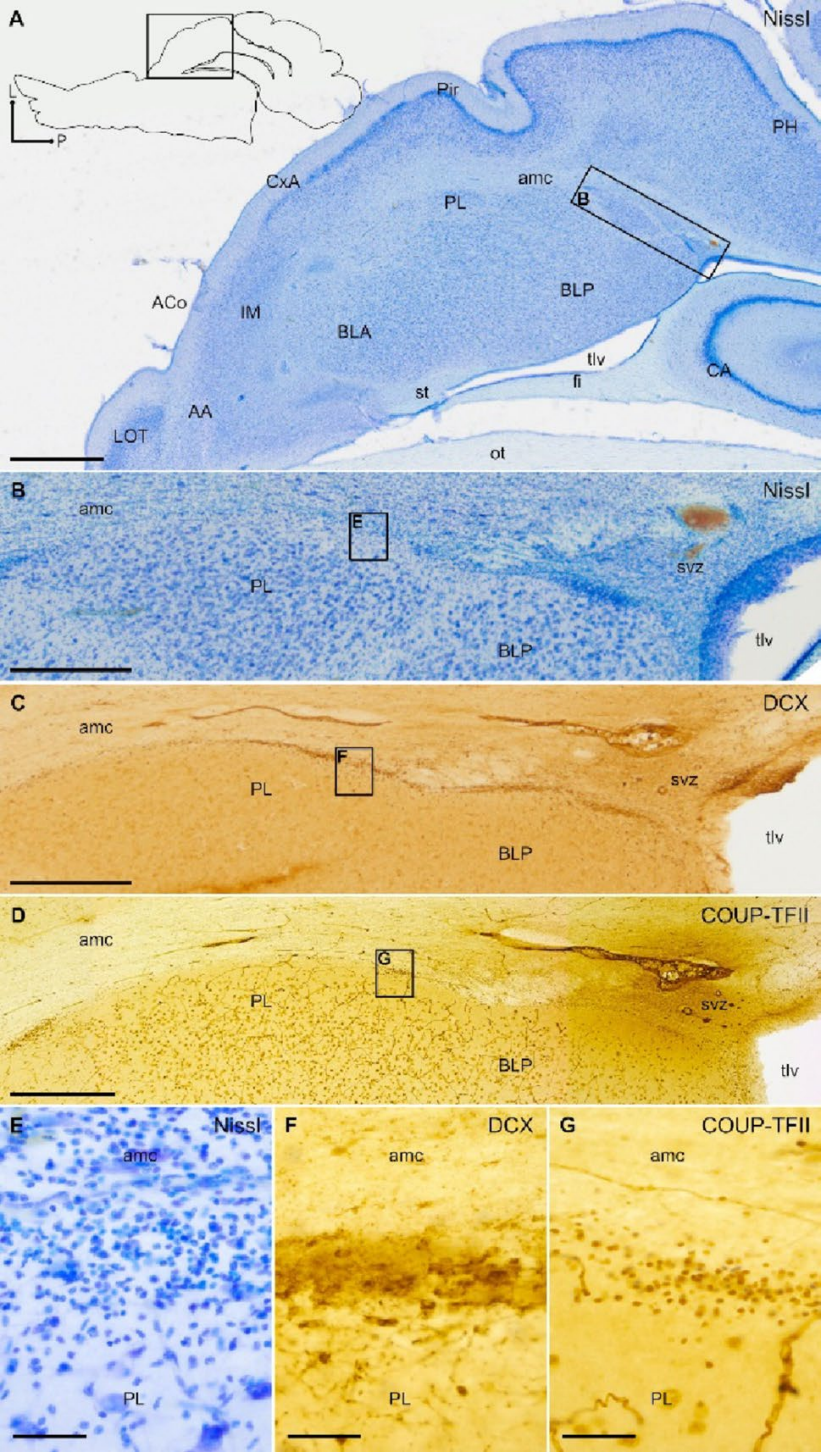
Horizontal sections showing migratory-like DCX + and COUP-TFII+ cells extending rostrally from the vz/svz along the amygdalar capsule (amc) and adjacent paralaminar nucleus (PL). **A**–**G** Parallel horizontal sections at a similar level of the pallial amygdala and its related posterior vz/svz, adjacent to the temporal horn of the lateral ventricle (tlv), stained for Nissl (**A**) or immunohistochemistry for DCX (**C**) or COUP-TFII (**D**). Squared area in A is shown at higher magnification in B. Squared areas in B, C, and D are shown at higher magnification in E, F, and G respectively. (**B**, **C**, **D**) Detail of the vz/svz and its continuation through the inner part of the amc, adjacent to the paralaminar nucleus (PL) and other parts of the basal amygdalar complex (BCA, including BLA and BLP). Clusters of small cells immunoreactive for DCX (C) and COUP-TFII (**D**) can be detected along the inner part of amc and adjacent PL. An increasing gradient of these clusters can be detected throughout the anteroposterior axis, being more abundant in the posterior part of the amc (near the svz) and scarcer in its anterior parts. **E**–**G** Detail of a cluster with some migratory-like cells with small, round somas expressing DCX (**F**) and COUP-TFII (**G**). A schematic representation of the horizontal section, and the anteroposterior and lateromedial axes are shown in A for orientation. For other abbreviations, see list. Scales: A = 2 mm; B, C, D = 500 μm; E, F, G = 50 μm

Moreover, from this hotspot of the vz/svz, we found chains of radially oriented migratory-like DCX+ cells, coexpressing COUP-TFII, that were in continuity with the cell clusters of lateral PL (Figs. 6 and 8). Thus, this particular posterolateral and inferior sector of vz/svz might be the source of the DCX+ immature neurons found in lateral PL. The horizontal plane was the best to follow this continuity from the vz/svz to the PL clusters (Figs. 6 and 8), and showed that the chains of migratory-like DCX+ cells in continuity with the DCX+/COUP-TFII+ cell clusters of PL occupied an inner position in the amygdalar capsule (Figs. 6 and 8), while other DCX+ cell chains located more superficially did not overlap nor coexpress COUP-TFII and appeared to be related to the endopiriform/piriform region.

**Fig. 9:**
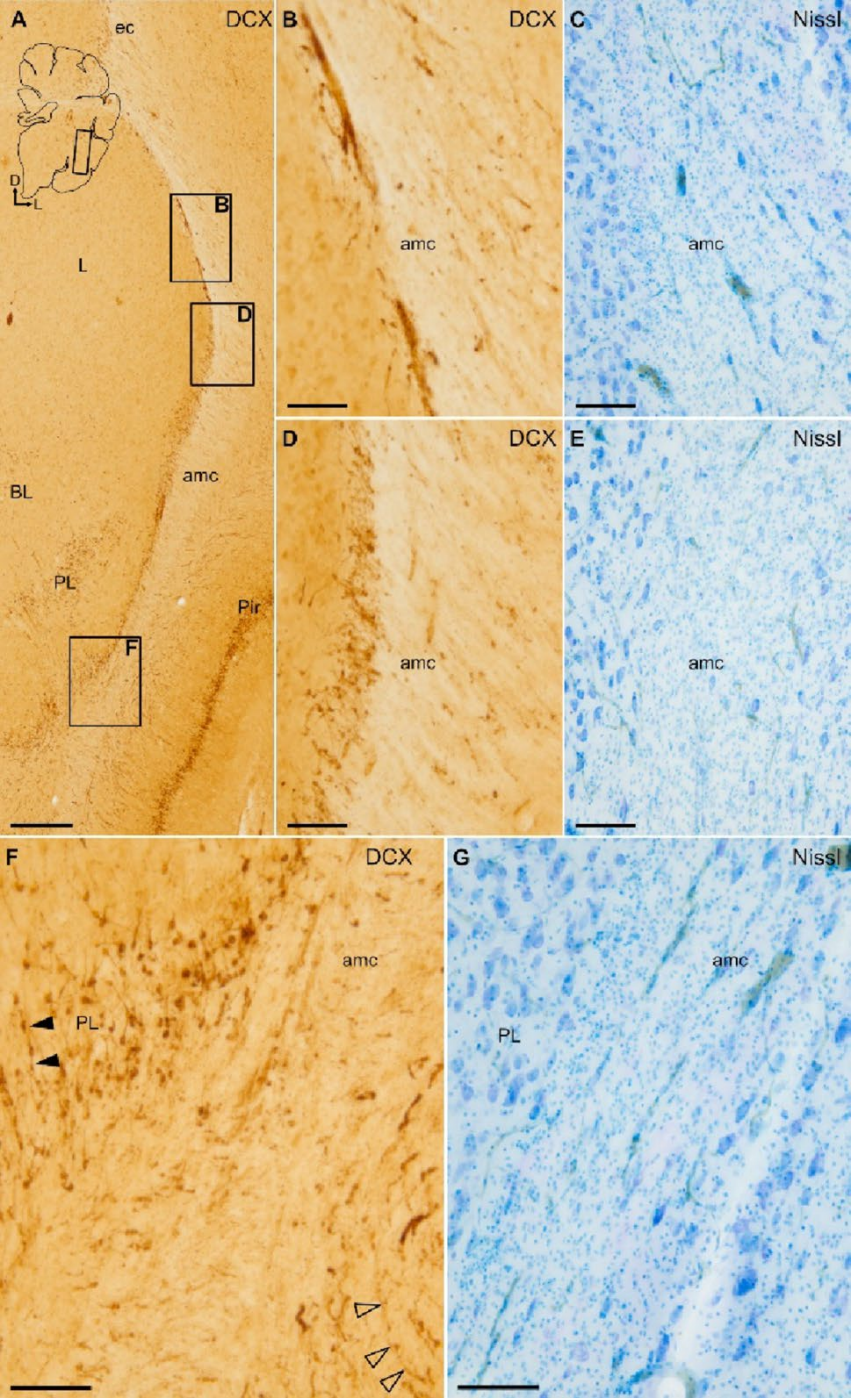
Frontal sections showing migratory-like DCX+ cells through the amygdalar capsule (amc) and adjacent paralaminar nucleus (PL). **A**–**G** Parallel frontal sections (similar level to that in Fig. 1) stained for immunohistochemistry for DCX (**A**, details in **B**, **D**, **F**) or Nissl (**C**, **E**, **G**), showing DCX+ cells in the inner part of amc and adjacent PL. Squared areas in A are shown at higher magnification in B, D, and F. **B** Detail of dorsal part of the amc, adjacent to the lateral nucleus (**L**) of the basal amygdalar complex (BCA), showing small DCX+ cells with a migratory-like morphology. **D**, **F** Detail of DCX immunoreactive cells in more ventral parts of the amc, some having a migratory-like morphology with the leading process oriented toward the PL clusters. Other DCX+ cells are organized in chains oriented toward PL (filled arrowheads in **F**). In the external part of the amc (**F**), DCX+ cells show the opposite orientation, toward the endopiriform nuclei and the piriform cortex (empty arrowheads in **F**). Schematic representation of the frontal section, and the mediolateral and dorsoventral axes are shown in A for orientation. For other abbreviations, see list. Scales: A = 500 μm; B, C, D, E, F, G = 100 μm

**Fig. 10:**
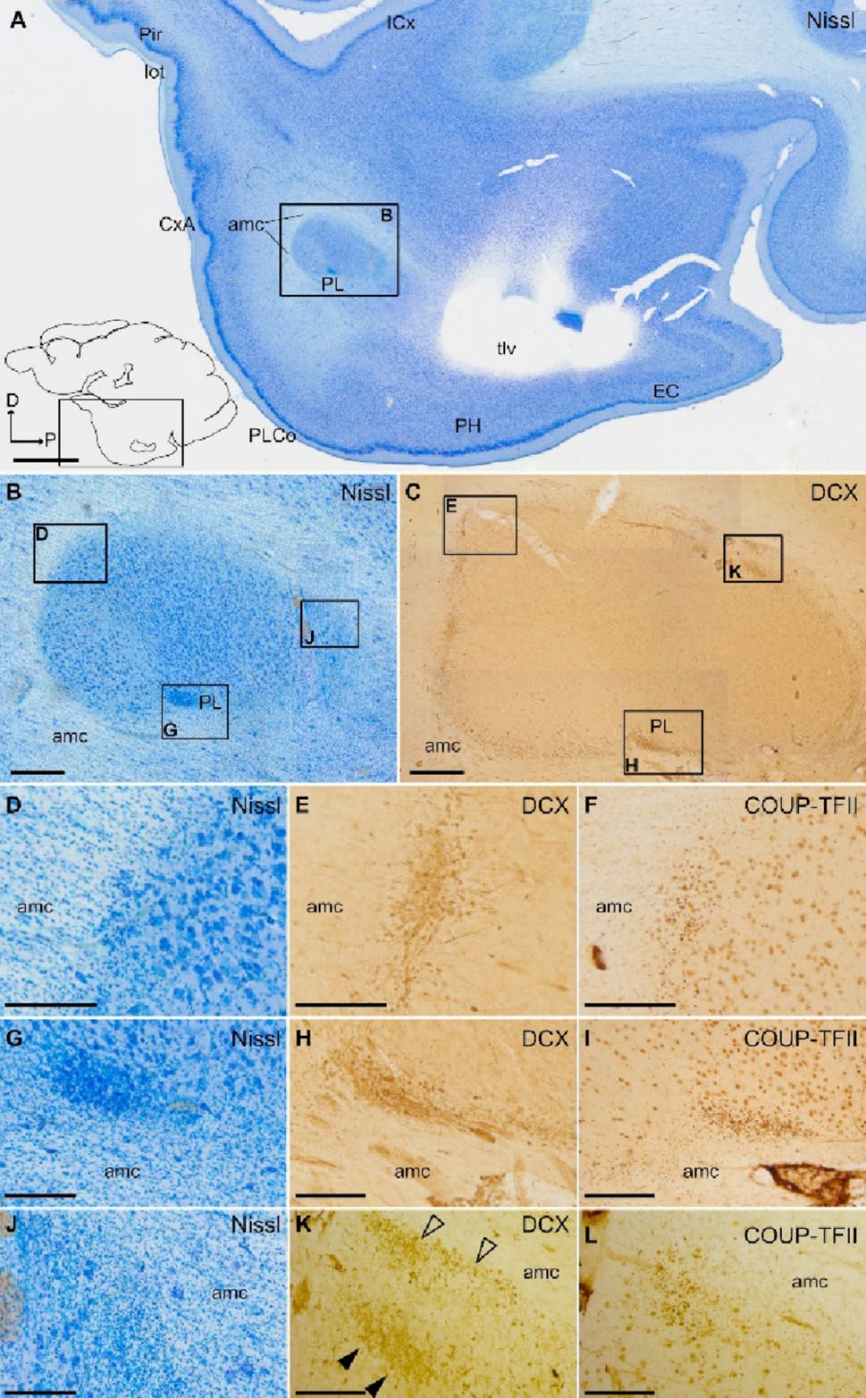
Sagittal sections showing DCX + and COUP-TFII+ cells through the amygdalar capsule (amc) and adjacent paralaminar nucleus (PL). **A**–**L** Parallel sagittal sections or their details at similar lateral level stained for Nissl (**A**, details in **B**, **D**, **G**, **J**) or immunohistochemistry for DCX (**C**, details in **E**, **H**, **K**) or COUP-TFII (**F**, **I**, **L**), showing labeled cells in the amygdalar capsule (amc) and adjacent paralaminar nucleus. Note the small size of the cells and the overlapping position of DCX + and COUP-TFII+ labeling. Squared area in A is shown at higher magnification in **B**. Squared areas in B are shown at higher magnification in **D**, **G**, and **J**. Squared areas in C are shown at higher magnification in E, H, and K. **B**, **C** Detail of the amc laterally and ventrally surrounding the basal magnocellular complex (BCA), stained for Nissl (**B**) and immunohistochemically processed for DCX (**C**). (**D**–**F**, **G**–**I**, and **J**–**L**) Details showing groups of DCX + and COUP-TFII+ cells with small, round or fusiform somas in the inner border of amc (empty arrowheads in **K**), some of which are oriented toward the clusters of labeled cells in the adjacent PL (pointed with filled arrowheads in **K**). Schematic representation of the sagittal section, and the anteroposterior and dorsoventral axes are shown in A for orientation. For other abbreviations, see list. Scales: A, B, C = 500 μm; D, E, F, G, H, I, J, K, L = 200 μm

The continuity of DCX+ cell chains from the posterolateral inferior vz/svz to the PL cell clusters could not be observed in the frontal (Fig. 9) of sagittal planes (Fig. 10), although in these section planes it was possible to observe short chains of DCX+ cells in the inner part of the amygdalar capsule, which appeared to reach the lateral PL clusters (Figs. 9A, B,F; filled arrowheads in F; 10E, H,K; empty arrowheads in K). In contrast, DCX+ cell chains of the external part of the amygdalar capsule were oriented toward the endopiriform/piriform region (empty arrowheads in Figs. 9F empty arrowheads).

Finally, we observed tangentially oriented chains of DCX+ cells, aligned parallel to the ventricular surface, that appear to separate from the same posterolateral and inferior vz/svz rich in small cells immunoreactive for DCX and COUP-TFII mentioned above (Fig. 6E-F’’). These tangentially oriented cell chains might contribute to at least part of the DCX+ cells observed in medial PL, but we cannot discard that other immature cells might be produced in the vz/svz sector adjacent to medial PL, since we also observed a few cases of DCX/Ki-67 coexpression there (Fig. 7B-B’’).

## Discussion

Our results on DCX expression showed prolonged postnatal plasticity in the swine pallial amygdala that extends at least until juvenile ages, which is comparable to the situation described in primates (De Campo and Fudge 2012; Sorrells et al. 2019; Ghibaudi et al. 2025). As previously shown in primates (De Campo and Fudge 2012; Sorrells et al. 2019; Ghibaudi et al. 2025), in swine this protracted plasticity of the amygdala is associated to the presence of immature cells in the PL, which we identified as a subdivision located along the external border of BCA, rich in immature DCX+ cells, most of which express NeuN (indicating a neuronal phenotype) and the transcription factor COUP-TFII / NR2F2, but not FOXP2 (which identified the intercalated amygdalar cells). In primates, it has been proposed that the PL could serve as a reservoir to provide new neurons for the basal (basolateral) and accessory (basomedial) nuclei of BCA (Chareyron et al. 2012; Avino et al. 2018; Sorrells et al. 2019). As in primates, the swine PL could serve as a reservoir of immature neurons for the pallial amygdala. However, the PL seems to be a different nucleus of BCA, containing neurons with different maturation degree as suggested by the shell-like organization of DCX+ cell clusters around non-DCX cells with mature morphology (Fig. 1). Therefore, most immature cells of PL might continue residing within this nucleus upon maturation, although we cannot discard the possibility that a few migrate to other BCA nuclei.

**Fig. 11:**
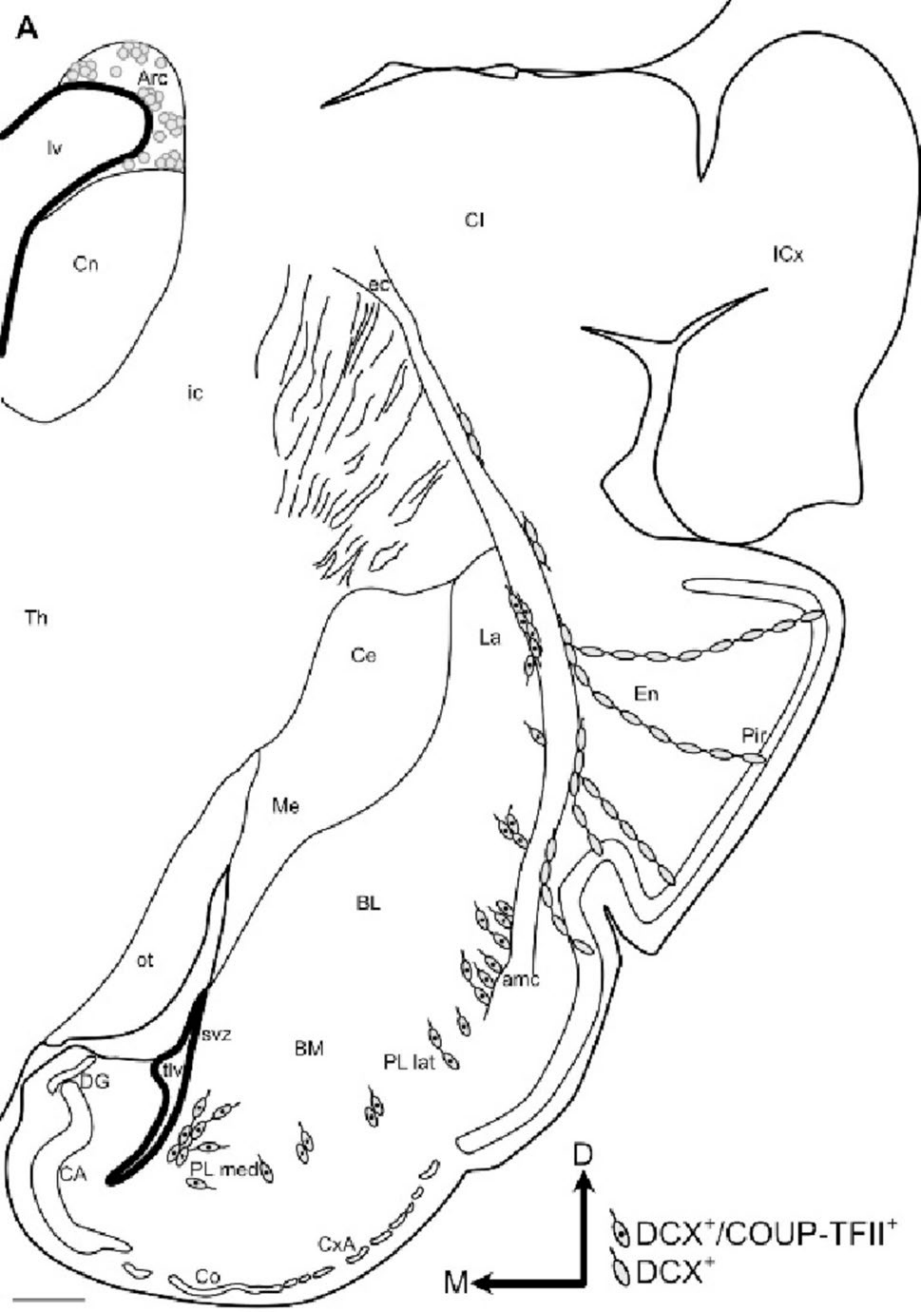
Schematic drawing of a frontal section showing the possible origin, migratory pathways and distribution of immature DCX + and DCX+/COUP-TFII+ neurons in the pallial amygdala of juvenile swine. **A** Schematic representation of a frontal section of juvenile swine brain at the level of the amygdala, showing DCX + and DCX+/COUP-TFII+ cells. Round cells in the ventricular/subventricular zones (vz/svz) represent possible sources of immature cells, while elongated somas represent the migratory-like cells found along the external (ec) and amygdalar capsules (amc), plus the adjacent areas of the endopiriform/piriform region and the basal complex of the amygdala (BCA), including the paralaminar nucleus (PL). Single labeled DCX+ cells are drawn as empty circles (such as those in the vz/svz of the Arc) or empty fusiform cells (as those in ec, the external part of amc, and those found in the endopiriform/piriform region). In contrast, double labeled cells immunoreactive for DCX and COUP-TFII are represented with a black dot in the center, indicating the COUP-TFII immunoreactive cell nucleus, and are found in the inner part of amc and lateral and medial subdivisions of PL (PL lat, PL med). Since DCX+ cells of the Arc do not express COUP-TFII, it is unlikely they produce the DCX+/COUP-TFII+ immature cells of the pallial amygdala. Thus, as previously shown, the Arc might mainly produce immature cells for the olfactory bulb and anterior olfactory areas (through the rostral migratory stream) and the olfactory structures of the piriform lobe (through ec and external parts of amc). Dorsoventral and mediolateral axes are indicated for orientation. For other abbreviations, see list. Scales: A = 1 mm

**Fig. 12:**
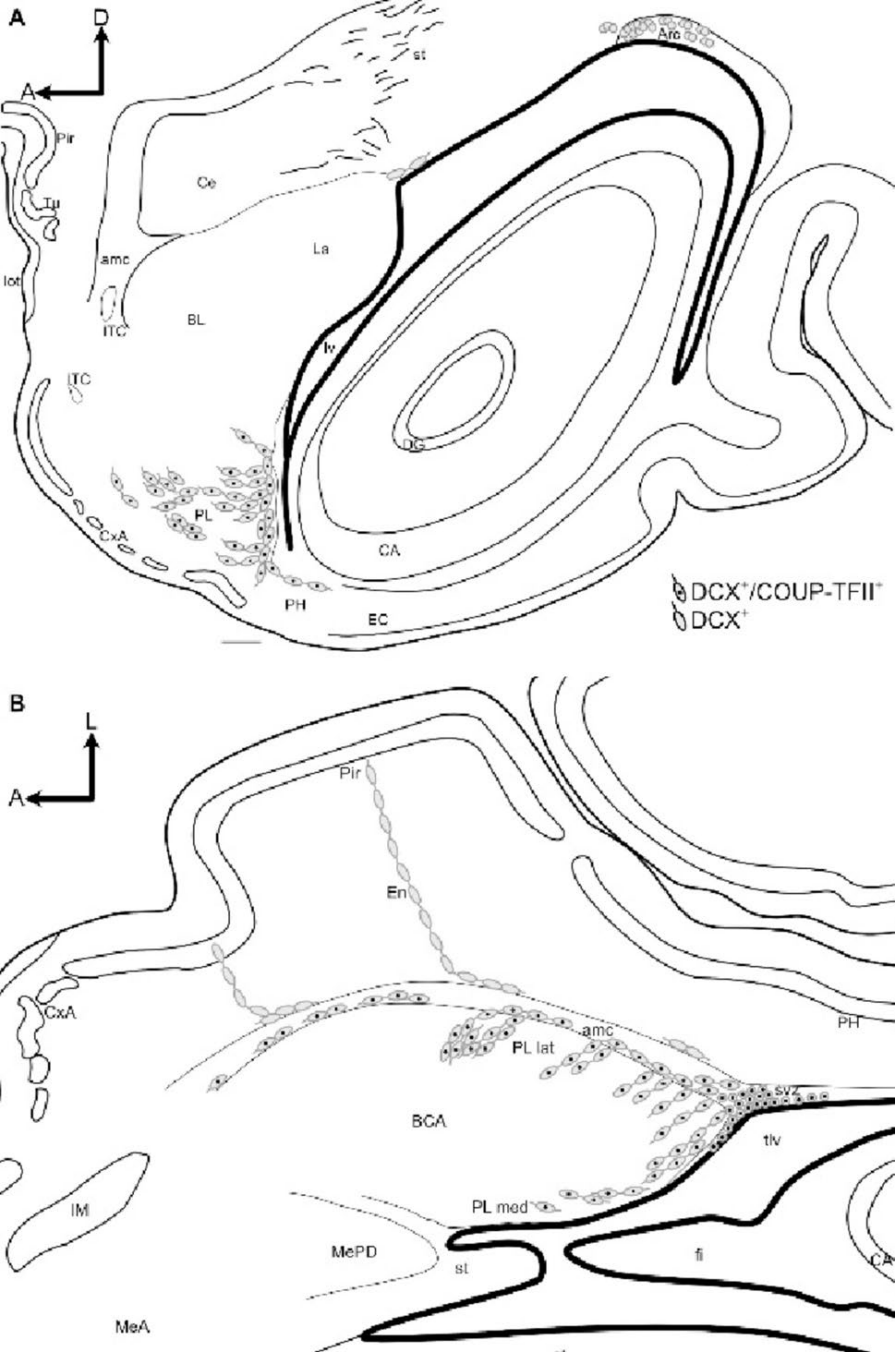
Schematic drawings of sagittal and horizontal sections showing the possible origin, migratory pathways and distribution of immature DCX + and DCX+/COUP-TFII+ neurons in the pallial amygdala of juvenile swine. **A**, **B** Schematic representation of sagittal (A) and horizontal (B) sections from juvenile swine brain, at the level of the pallial amygdala, showing DCX + and DCX+/COUP-TFII+ cells. Round cells in the ventricular/subventricular zones (vz/svz) represent possible sources of immature cells, while elongated somas represent the migratory-like cells found along the external (ec) and amygdalar capsules (amc), plus the adjacent areas of the endopiriform/piriform region and the basal complex of the amygdala (BCA), including the paralaminar nucleus (PL). Single labeled DCX+ cells are drawn as empty circles (such as those in the vz/svz of the Arc) or empty fusiform cells (as those in ec, the external part of amc, and those found in the endopiriform/piriform region). In contrast, double labeled cells immunoreactive for DCX and COUP-TFII are drawn with a black dot in the center, representing the COUP-TFII immunoreactive cell nucleus. Double labeled cells are found in the posterolateral inferior vz/svz, as well as in the inner part of amc and adjacent PL. This posterolateral and inferior vz/svz sector rich in double-labeled cells is likely the origin of the double-labeled immature cells found in the inner part of amc, in PL and other parts of the pallial amygdala. Some cells might also migrate from this vz/svz sector to adjacent parahippocampal areas (PH). In contrast, the Arc only contains single-labeled DCX+ cells, and thus it is unlikely that it is a source of immature cells for the pallial amygdala. Anteroposterior and mediolateral axes are indicated in A for orientation. Dorsoventral and anteroposterior axes are indicated in B for orientation. For other abbreviations, see list. Scales: A = 1 mm

Previous studies indicated that the size of the PL reservoir correlates with the degree of cortical expansion, being larger in gyrencephalic than in lissencephalic brains (Ghibaudi et al. 2025). Species with a gyrencephalic brain also have a relatively large pallial amygdala, which is particularly evident in the BCA (De Campo and Fudge 2012; Piumatti et al. 2018; Ghibaudi et al. 2025). Our results agree with this proposal, as the swine has a gyrencephalic brain, with large cortices and BCA relative to subpallial structures such as the striatum and central amygdala, and we also observed a well developed PL, including lateral and medial subdivisions (schemes in Figs. 11 and 12), as described in primates (De Campo and Fudge 2012). In contrast, lissencephalic rodents such as the mouse and rat, with smaller cortices and BCA, only have a rudimentary PL, which remained unnoticed or poorly described until recently (De Campo and Fudge 2012; Alderman et al. 2024).

A major distinctive feature of the immature DCX+ cells of PL is that they express COUP-TFII and several glutamatergic markers such as TBR1 and VGLUT2 (SCL17A6), but not GABAergic markers as SP8, GAD65 and GAD67 (Sorrells et al. 2019; Alderman et al. 2024). Moreover, deletion of COUP-TFII had a significant impact on excitatory populations (TBR1 and GLU2R) of the rodent BCA, while few effects were reported in inhibitory GAD67 + interneurons (Tang et al. 2012). In our material in swine, we found that a few DCX+ cells of PL express the transcription factor BRN2 (POU3F2), present in some subsets of immature glutamatergic cells (Dominguez et al. 2013; Brunjes and Osterberg 2015), such as many of those of the swine postnatal olfactory structures (Freixes et al. 2025). However, most DCX+ cells of swine PL did not express BRN2 (POU3F2), at least during juvenile postnatal ages, suggesting that at this age they express glutamatergic markers different to those found in immature neurons of olfactory structures.

We observed that many DCX+/COUP-TFII+ cells found in juvenile swine PL had an elongated, migratory-like morphology, which prompted us to investigate possible origin and migration routes by analyzing sections in different planes. We could follow the immature cells posteroventrally, until the vicinity of the temporal horn of the lateral ventricle, where we found a reservoir of DCX+/COUP-TFII+ immature cells in a posterolateral inferior sector of the vz/svz, from where cells appeared to migrate radially to lateral PL and tangentially to medial PL (schemes in Figs. 11 and 12). However, this proposal would require confirmation using cell migration assays. The vz/svz associated to PL appears to relate to the lateralmost progenitor domain of the pallial amygdala, and it has been identified previously in the lateral part of the “inferior ganglionic eminence” (De Campo and Fudge 2012), which is not a subpallial domain as the name suggests, but it really represents the progenitor zone of the pallial amygdala (García-Calero et al. 2020). Notably, many DCX + and COUP-TFII+ cells in this vz/svz sector expressed Ki-67 in juvenile swine, indicating that new neurons for the PL could be produced postnatally, at least until juvenile stages. This would agree with previous findings in primates (Bernier et al. 2002), including humans (Sorrells et al. 2019; Roeder et al. 2022), and with our results of the swine olfactory areas (Freixes et al. 2025). In addition, other DCX+/COUP-TFII+ cells found in this vz/svz sector and the associated PL might represent quiescent immature neurons that were generated previously, as suggested in adult animals of different mammalian species (Ghibaudi et al. 2025).

In contrast to glutamatergic COUP-TFII neurons of PL that appear to be produced in the amygdalar pallium (as discussed above), GABAergic COUP-TFII cells are produced during development in the caudal ganglionic and part of the medial ganglionic eminences of the subpallium, and they constitute a source of interneurons that migrate tangentially to the cortex (Tang et al. 2012; Alderman et al. 2024; Kim et al. 2025). Remnants of GABAergic immature neurons continue to be produced postnatally from the vz/svz of the Arc, at least in human and non-human primate infants and in two-days-old piglets (Kim et al. 2025). This could be the source of the COUP-TFII+/SP8 + cells found in the cerebral cortex and the piriform cortex. However, by using single-cell transcriptome of different rostrocaudal parts of the Arc, Kim et al. (2025) also identified that the vz/svz of the Arc gives rise to a temporo-ventral migratory stream of DCX+ cells that express both COUP-TFII and TBR1, suggesting that cells of this stream are glutamatergic. Many of these cells appear to migrate to the temporal cortex, but some were also found in the piriform cortex (Kim et al. 2025). It is unclear whether the latter temporo-ventral stream of DCX+/COUP-TFII+/TBR1 + cells includes part of the PL-associated vz/svz cells. In our juvenile swine data, we found that the Arc does not express COUP-TFII, but we did not carry out a systematic analysis of all levels of Arc. More studies will be needed to clarify this issue.

Compared to that in other mammals, the density of DCX+ immature cells in the pallial amygdala, concentrated in PL, is not only high in gyrencephalic human and non-human primates, but also in small lissencephalic primates as the marmoset (Ghibaudi et al. 2025). Thus, primates stand above other mammals regarding pallial amygdala plasticity, independent of the secondary loss of gyri as it appears to occur in marmosets (Kelava et al. 2013). In other mammalian radiations, gyrencephalic brains have often been associated with high levels of non-neurogenic postnatal plasticity (Piumatti et al. 2018; Freixes et al. 2025; Ghibaudi et al. 2025). However, this prolonged plasticity and the location of the immature cells in the brain need to be understood in the context of the ecological adaptations of each species: for example, the high postnatal plasticity in the olfactory areas of swine could be related to the relevance of olfactory cues for feeding and social interactions in these animals (Brunjes et al. 2016; also discussed by Freixes et al. 2025). Regarding the high postnatal plasticity found in the pallial amygdala, what could be the advantage of having a prolonged production of new neurons in this brain structure? In primates, the BCA is the part of the amygdala more broadly connected to the neocortex (Ghashghaei and Barbas 2002; Ghashghaei et al. 2007; Pessoa 2008). Having a large reservoir of immature neurons in this nuclear complex may be required for achieving a more sophisticated regulation of emotional learning (Jin et al. 2015; Kim et al. 2017; discussed by Sorrell et al. 2019), as well as a more plastic adaptation to ever-changing complex social contexts (Chareyron et al. 2012; Avino et al. 2018; Ghibaudi et al. 2025).

Overall, our data, together with other studies (Kostović et al. 2019; Liu et al. 2021), highlight the similarities between swine and humans in terms of brain anatomy and ontogeny, including a protracted development of the pallial amygdala. Since the incorporation of immature neurons in the human PL at juvenile ages seems to be dysfunctional in autism spectrum disorder (Avino et al. 2018), the swine emerges as a good animal model to further study postnatal plasticity in functional and dysfunctional contexts.

## Supplementary Information

Below is the link to the electronic supplementary material.

Supplementary Material 1 Supplementary Fig. 1. Validation of primary antibodies by western blotting. Western blot analysis of DCX and COUP-TFII antibodies in swine brain tissue. (A) Western blot of goat anti-doublecortin antibody (Santa Cruz Biotechnologies, sc-8066) with samples of juvenile brain, taken from the caudate nucleus (Cn), the neocortex (NCx), the piriform cortex (Pir) and a homogenized whole-hemisphere of an E50 embryo. Results show a band of ~40kDa in the embryo brain tissue, consistent with that of DCX. (B) Western blot of rabbit anti-doublecortin antibody (Abcam, ab18723) with a sample from a homogenized whole-hemisphere of an E50 embryo, also showing a band of ~ 40kDa. (C) Western blot of mouse anti-COUP-TFII antibody (Bio-Techne R&D Systems, PP-H7147-00) with two samples of 20µg and 40 µg of protein from a homogenized whole-hemisphere of an E50 embryo, showing a band of ~ 55kDa, consistent with COUP-TFII.

Supplementary Material 2 Supplementary Fig. 2. Distribution of DCX+ clusters in the paralaminar nucleus (PL) of juvenile swine pallial amygdala. (A, B) Frontal sections at two different levels of the amygdala and (C) horizontal section at the level of the amygdala, immunohistochemically stained for DCX. (A) Anterior and (B) posterior frontal sections allow the visualization of DCX+ cell clusters in the PL, with and increasing gradient along the anteroposterior axis. DCX+ cells are found in lateral and medial subdivisions of PL (PL lat and PL med), but those in PL lat show a shell-like organization, around islands of non-stained cells (asterisks). (C) Horizontal section also shows DCX+ cells in the posterolateral inferior ventricular/subventricular zones (vz/svz), adjacent to the temporal horn of the lateral ventricle, as well as in the PL lat, which decrease as we move toward anterior parts of the pallial amygdala. Asterisks point to DCX+ cells with shell-like distribution in PL lat. Schematic representation of coronal sections and mediolateral and dorsoventral axes are shown in A and B for orientation. Schematic representation of the horizontal section and mediolateral and anteroposterior axes are shown in C for orientation. For abbreviations, see list. Scales: A, B, C = 2 mm.

Supplementary Material 3 Supplementary Fig. 3. Distribution of DCX+ cells in the Arc and through the rostral migratory stream, and their relation to COUP-TFII. (A-C, E-F, H-I) Sagittal sections at similar level (same level to that in Fig. 11), stained for Nissl (A) or immunohistochemistry for DCX (C, F, I). Note the presence of DCX+ cells in the ventricular/subventricular zones (vz/svz) of the Arc, adjacent to the dorsal part of the lateral ventricle, and in the rostral migratory stream (RMS) or in the external capsule (ec) adjacent to the putamen. Squared areas in A are shown at higher magnification in B, E and H. (B, C) Detail of the Arc, where DCX+ cells are grouped in clusters and form migratory-like chains entering the RMS. (D-D’’) Confocal images of a sagittal section (similar level to that in A) processed for double immunofluorescence for DCX (green) and COUP-TFII (magenta). Note the abundant DCX+ cells and their processes, but the lack of coexpression with COUP-TFII. (E, F) Detail of the DCX+ cell patches along the ec, adjacent to the putamen (Pu), with processes oriented along the anteroposterior axis (F). (G-G’’) Confocal images of a sagittal section (similar level to that in A) processed for double immunofluorescence for DCX (green) and COUP-TFII (magenta), showing DCX+ cells in ec (G’), but none of them coexpresses COUP-TFII (G’’). (H, I) Detail of the rostral part of the ec, adjacent to the RMS, where DCX+ elongated cells align along the anteroposterior axis. (J-J’’) Confocal images of a sagittal section (similar level to that in A) processed for double immunofluorescence for DCX (green) and COUP-TFII (magenta), showing no coexpression of DCX and COUP-TFII in migratory-like chains of the RMS. Schematic representation of the sagittal section and the anteroposterior and dorsoventral axes are shown in A for orientation. For other abbreviations, see list. Scales: A = 2 mm; B, E, H = 250 μm (also applies to C, F and I); D, G, J = 100 μm (also applies to D’-D’’, G’-G’’ and J’-J’’).

## Data Availability

The most relevant data are included in the Figures of this article. Additional data is available upon request and agreement.

## Abbreviations

A: Anterior
AA: Anterior amygdalar area
ABC: Avidin–biotin complex
ac: Anterior commissure
ACo: Anterior cortical amygdalar area
AHi: Amygdalohippocampal area
amc: Amygdalar capsule
AO: Anterior olfactory area
BCA: Basal complex of the amygdala
BCL2: B cell lymphoma 2 protein
BL: Basolateral nucleus of the basal complex of the amygdala
BLA: Basolateral nucleus of the basal complex of the amygdala, anterior part
BLP: Basolateral nucleus of the basal complex of the amygdala, posterior part
BM: Basomedial nucleus of the basal complex of the amygdala
Brn2 (Pou3f2): POU class 3 homeobox 2
BSA: Bovine serum albumin
CA: Cornu ammonis
Ce: Central nucleus of the amygdala
Cl: Claustrum
Cn: Caudate nucleus
Co: Cortical amygdalar area
COUP-TFII (NR2F2): Chicken ovalbumin upstream promoter transcription factor II
CxA: Cortex-amygdala transition zone
D: Dorsal
DCX: Doublecortin
DG: Dentate gyrus
EC: Entorhinal cortex
ec: External capsule
En: Endopiriform nucleus
fi: Fimbria
FOXP2: Forkhead box P2 transcription factor
fx: Fornix
GP: Globus pallidus
Gt: Goat
HF: Hippocampal formation
HRP: Horseradish peroxidase conjugated secondary antibodies
ic: Internal capsule
ICx: Insular cortex
IF: Immunofluorescence
IHC: Immunohistochemistry
IM: Intercalated amygdalar nucleus, main part
ITC: Intercalated cells of the amygdala
IV: Intravenous
L: Lateral
La: Lateral nucleus of the basal complex of the amygdala
lot: Lateral olfactory tract
LOT: Nucleus of the lateral olfactory tract
lv: Lateral ventricle
Me: Medial nucleus of the amygdala
MeA: Medial nucleus of the amygdala, anterior part
MePD: Medial nucleus of the amygdala, posterodorsal part
Ms: Mouse
NeuN: Neuronal nuclear protein (also knows as FOX3)
ot: Optic tract
P: Posterior
PB: Phosphate buffer
PB-Tx: Phosphate buffer containing Triton-X 100
PFA: Paraformaldehyde
PH: Parahippocampal area
Pir: Piriform nucleus
PL: Paralaminar nucleus
PL lat: Paralaminar nucleus, lateral subdivision
PL med: Paralaminar nucleus, medial subdivision
PLCo: Posterolateral cortical amygdalar area
PMCo: Posteromedial cortical amygdalar area
PSA-NCAM: Polysialylated neural cell adhesion molecule
PTh: Prethalamus
Pu: Putamen
Rb: Rabbit
RIPA: Radioimmunoprecipitation assay
RMS: Rostral migratory stream
Rt: Reticular nucleus of the prethalamus
S: Subiculum
SP8: Specificity protein 8 (SP8 transcription factor)
st: Stria terminalis
SVZ/svz: Subventricular zone
Tbr1: T-box brain transcription factor 1
TBST: Tris-buffered saline containing Tween 20
Th: Thalamus
tlv: Temporal horn of the lateral ventricle
Tu: Olfactory tubercle
VGLUT2: Vesicular glutamate transporter 2
VZ/vz: Ventricular zone
WB: Western blot
