# Supplementary figures and images for: Postnatal plasticity in the paralaminar nucleus of the pallial amygdala in juvenile swine brain
Source: Brain Struct Funct. 2026 Apr 6;231(3):51. doi: 10.1007/s00429-026-03106-8 (PMC13050766; doi:10.1007/s00429-026-03106-8)

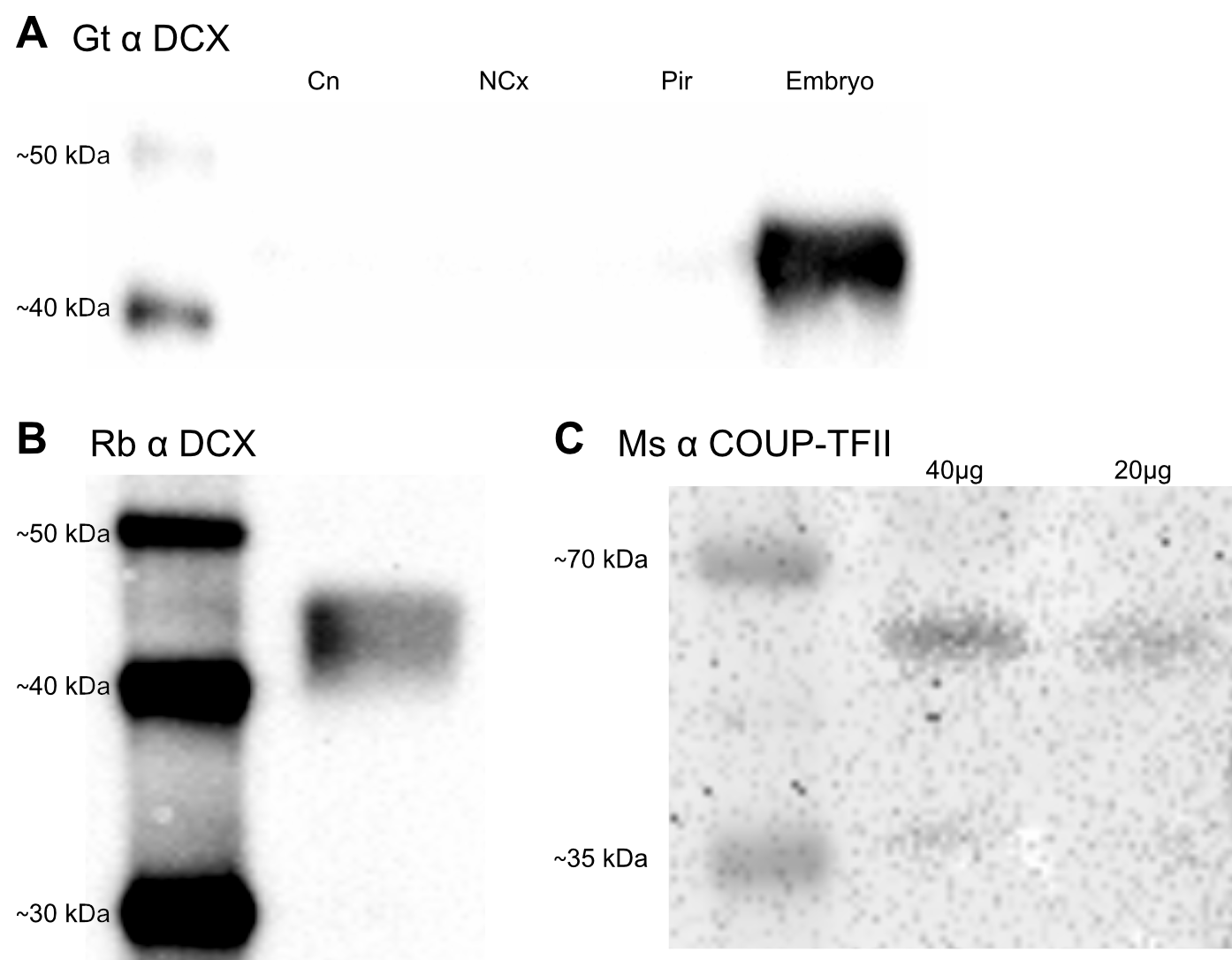

Supplement: Supplementary file 1 — Supplementary Material 1 Supplementary Fig. 1. Validation of primary antibodies by western blotting. Western blot analysis of DCX and COUP-TFII antibodies in swine brain tissue. (A) Western blot of goat anti-doublecortin antibody (Santa Cruz Biotechnologies, sc-8066) with samples of juvenile brain, taken from the caudate nucleus (Cn), the neocortex (NCx), the piriform cortex (Pir) and a homogenized whole-hemisphere of an E50 embryo. Results show a band of ~40kDa in the embryo brain tissue, consistent with that of DCX. (B) Western blot of rabbit anti-doublecortin antibody (Abcam, ab18723) with a sample from a homogenized whole-hemisphere of an E50 embryo, also showing a band of ~ 40kDa. (C) Western blot of mouse anti-COUP-TFII antibody (Bio-Techne R&D Systems, PP-H7147-00) with two samples of 20µg and 40 µg of protein from a homogenized whole-hemisphere of an E50 embryo, showing a band of ~ 55kDa, consistent with COUP-TFII. [file 429_2026_3106_MOESM1_ESM.tiff]

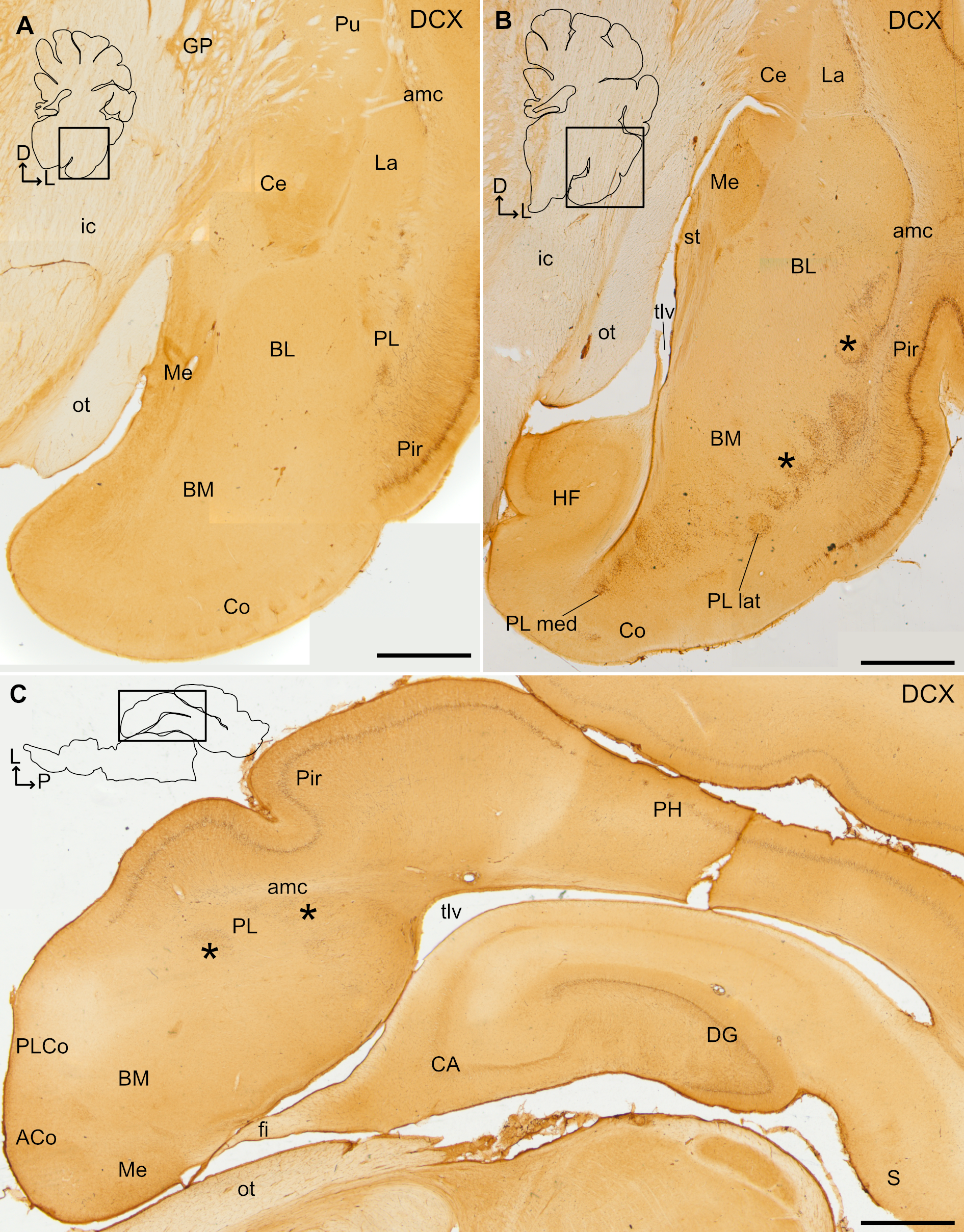

Supplement: Supplementary file 2 — Supplementary Material 2 Supplementary Fig. 2. Distribution of DCX+ clusters in the paralaminar nucleus (PL) of juvenile swine pallial amygdala. (A, B) Frontal sections at two different levels of the amygdala and (C) horizontal section at the level of the amygdala, immunohistochemically stained for DCX. (A) Anterior and (B) posterior frontal sections allow the visualization of DCX+ cell clusters in the PL, with and increasing gradient along the anteroposterior axis. DCX+ cells are found in lateral and medial subdivisions of PL (PL lat and PL med), but those in PL lat show a shell-like organization, around islands of non-stained cells (asterisks). (C) Horizontal section also shows DCX+ cells in the posterolateral inferior ventricular/subventricular zones (vz/svz), adjacent to the temporal horn of the lateral ventricle, as well as in the PL lat, which decrease as we move toward anterior parts of the pallial amygdala. Asterisks point to DCX+ cells with shell-like distribution in PL lat. Schematic representation of coronal sections and mediolateral and dorsoventral axes are shown in A and B for orientation. Schematic representation of the horizontal section and mediolateral and anteroposterior axes are shown in C for orientation. For abbreviations, see list. Scales: A, B, C = 2 mm. [file 429_2026_3106_MOESM2_ESM.tiff]

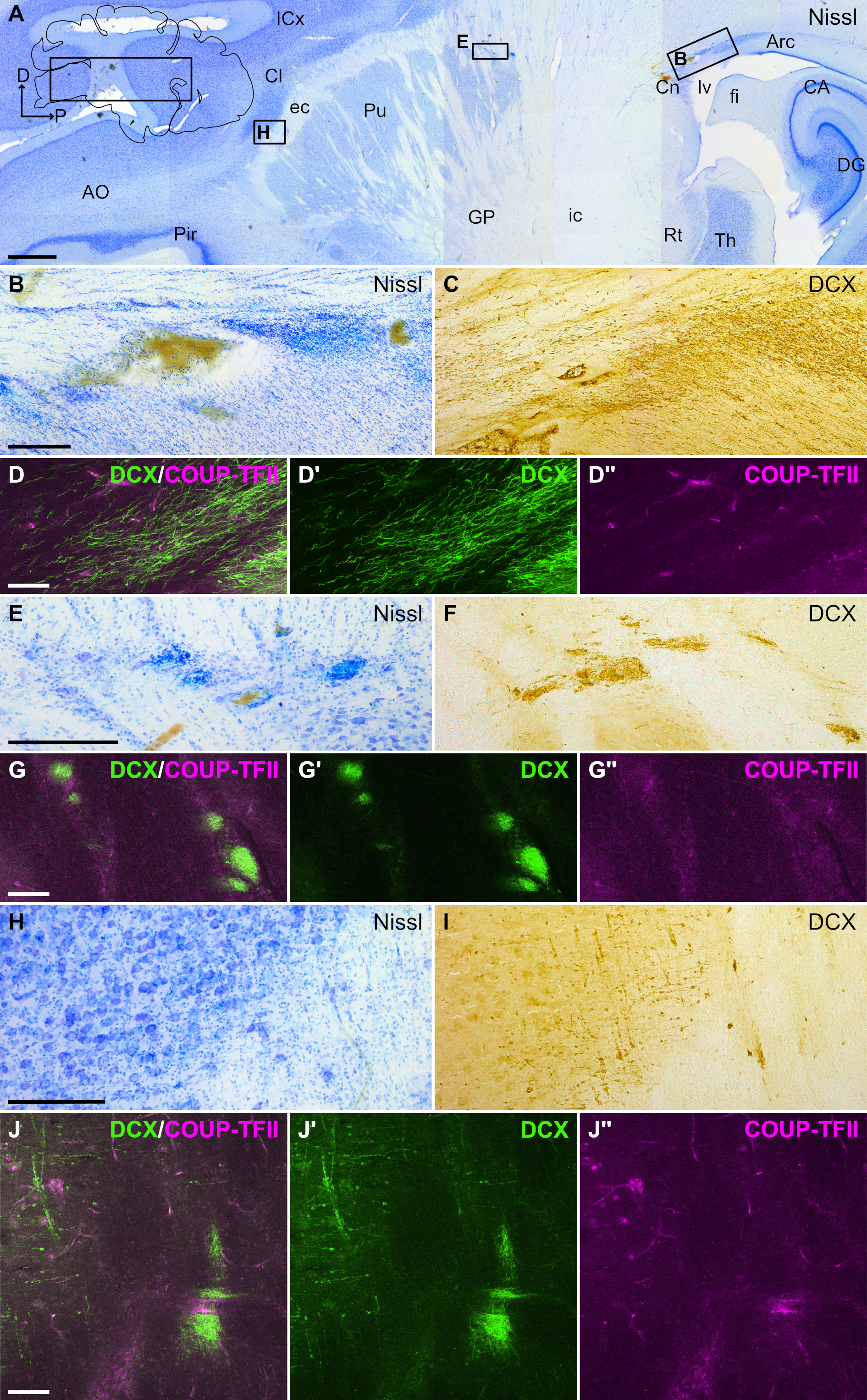

Supplement: Supplementary file 3 — Supplementary Material 3 Supplementary Fig. 3. Distribution of DCX+ cells in the Arc and through the rostral migratory stream, and their relation to COUP-TFII. (A-C, E-F, H-I) Sagittal sections at similar level (same level to that in Fig. 11), stained for Nissl (A) or immunohistochemistry for DCX (C, F, I). Note the presence of DCX+ cells in the ventricular/subventricular zones (vz/svz) of the Arc, adjacent to the dorsal part of the lateral ventricle, and in the rostral migratory stream (RMS) or in the external capsule (ec) adjacent to the putamen. Squared areas in A are shown at higher magnification in B, E and H. (B, C) Detail of the Arc, where DCX+ cells are grouped in clusters and form migratory-like chains entering the RMS. (D-D’’) Confocal images of a sagittal section (similar level to that in A) processed for double immunofluorescence for DCX (green) and COUP-TFII (magenta). Note the abundant DCX+ cells and their processes, but the lack of coexpression with COUP-TFII. (E, F) Detail of the DCX+ cell patches along the ec, adjacent to the putamen (Pu), with processes oriented along the anteroposterior axis (F). (G-G’’) Confocal images of a sagittal section (similar level to that in A) processed for double immunofluorescence for DCX (green) and COUP-TFII (magenta), showing DCX+ cells in ec (G’), but none of them coexpresses COUP-TFII (G’’). (H, I) Detail of the rostral part of the ec, adjacent to the RMS, where DCX+ elongated cells align along the anteroposterior axis. (J-J’’) Confocal images of a sagittal section (similar level to that in A) processed for double immunofluorescence for DCX (green) and COUP-TFII (magenta), showing no coexpression of DCX and COUP-TFII in migratory-like chains of the RMS. Schematic representation of the sagittal section and the anteroposterior and dorsoventral axes are shown in A for orientation. For other abbreviations, see list. Scales: A = 2 mm; B, E, H = 250 μm (also applies to C, F and I); D, G, J = 100 μm (also applies to D’-D [file 429_2026_3106_MOESM3_ESM.tiff]
